# Single-cell profiling reveals a potent role of quercetin in promoting hair regeneration

**DOI:** 10.1093/procel/pwac062

**Published:** 2022-11-30

**Authors:** Qian Zhao, Yandong Zheng, Dongxin Zhao, Liyun Zhao, Lingling Geng, Shuai Ma, Yusheng Cai, Chengyu Liu, Yupeng Yan, Juan Carlos Izpisua Belmonte, Si Wang, Weiqi Zhang, Guang-Hui Liu, Jing Qu

**Affiliations:** Advanced Innovation Center for Human Brain Protection and National Clinical Research Center for Geriatric Disorders, Xuanwu Hospital Capital Medical University, Beijing 100053, China; Aging Translational Medicine Center, International Center for Aging and Cancer, Beijing Municipal Geriatric Medical Research Center, Xuan Wu Hospital, Capital Medical University, Beijing 100053, China; State Key Laboratory of Stem Cell and Reproductive Biology, Institute of Zoology, Chinese Academy of Sciences, Beijing 100101, China; University of Chinese Academy of Sciences, Beijing 100049, China; Shanghai Institute of Materia Medica, Chinese Academy of Sciences, Shanghai 201203, China; Advanced Innovation Center for Human Brain Protection and National Clinical Research Center for Geriatric Disorders, Xuanwu Hospital Capital Medical University, Beijing 100053, China; Aging Translational Medicine Center, International Center for Aging and Cancer, Beijing Municipal Geriatric Medical Research Center, Xuan Wu Hospital, Capital Medical University, Beijing 100053, China; Advanced Innovation Center for Human Brain Protection and National Clinical Research Center for Geriatric Disorders, Xuanwu Hospital Capital Medical University, Beijing 100053, China; Aging Translational Medicine Center, International Center for Aging and Cancer, Beijing Municipal Geriatric Medical Research Center, Xuan Wu Hospital, Capital Medical University, Beijing 100053, China; State Key Laboratory of Membrane Biology, Institute of Zoology, Chinese Academy of Sciences, Beijing 100101, China; Institute for Stem Cell and Regeneration, Chinese Academy of Sciences, Beijing 100101, China; Beijing Institute for Stem Cell and Regenerative Medicine, Beijing 100101, China; State Key Laboratory of Membrane Biology, Institute of Zoology, Chinese Academy of Sciences, Beijing 100101, China; Institute for Stem Cell and Regeneration, Chinese Academy of Sciences, Beijing 100101, China; Beijing Institute for Stem Cell and Regenerative Medicine, Beijing 100101, China; State Key Laboratory of Stem Cell and Reproductive Biology, Institute of Zoology, Chinese Academy of Sciences, Beijing 100101, China; University of Chinese Academy of Sciences, Beijing 100049, China; State Key Laboratory of Membrane Biology, Institute of Zoology, Chinese Academy of Sciences, Beijing 100101, China; Institute for Stem Cell and Regeneration, Chinese Academy of Sciences, Beijing 100101, China; Beijing Institute for Stem Cell and Regenerative Medicine, Beijing 100101, China; Altos Labs, Inc., San Diego, CA 94022, USA; Advanced Innovation Center for Human Brain Protection and National Clinical Research Center for Geriatric Disorders, Xuanwu Hospital Capital Medical University, Beijing 100053, China; Aging Translational Medicine Center, International Center for Aging and Cancer, Beijing Municipal Geriatric Medical Research Center, Xuan Wu Hospital, Capital Medical University, Beijing 100053, China; The Fifth People’s Hospital of Chongqing, Chongqing 400062, China; University of Chinese Academy of Sciences, Beijing 100049, China; Institute for Stem Cell and Regeneration, Chinese Academy of Sciences, Beijing 100101, China; Beijing Institute for Stem Cell and Regenerative Medicine, Beijing 100101, China; CAS Key Laboratory of Genomic and Precision Medicine, Beijing Institute of Genomics, Chinese Academy of Sciences, and China National Center for Bioinformation, Beijing 100101, China; Sino-Danish College, University of Chinese Academy of Sciences, Beijing 101408, China; Advanced Innovation Center for Human Brain Protection and National Clinical Research Center for Geriatric Disorders, Xuanwu Hospital Capital Medical University, Beijing 100053, China; Aging Translational Medicine Center, International Center for Aging and Cancer, Beijing Municipal Geriatric Medical Research Center, Xuan Wu Hospital, Capital Medical University, Beijing 100053, China; University of Chinese Academy of Sciences, Beijing 100049, China; State Key Laboratory of Membrane Biology, Institute of Zoology, Chinese Academy of Sciences, Beijing 100101, China; Institute for Stem Cell and Regeneration, Chinese Academy of Sciences, Beijing 100101, China; Beijing Institute for Stem Cell and Regenerative Medicine, Beijing 100101, China; State Key Laboratory of Stem Cell and Reproductive Biology, Institute of Zoology, Chinese Academy of Sciences, Beijing 100101, China; University of Chinese Academy of Sciences, Beijing 100049, China; Institute for Stem Cell and Regeneration, Chinese Academy of Sciences, Beijing 100101, China; Beijing Institute for Stem Cell and Regenerative Medicine, Beijing 100101, China

**Keywords:** single-cell RNA-sequencing, Que, hair follicle regeneration, endothelial cells, HIF-1α

## Abstract

Hair loss affects millions of people at some time in their life, and safe and efficient treatments for hair loss are a significant unmet medical need. We report that topical delivery of quercetin (Que) stimulates resting hair follicles to grow with rapid follicular keratinocyte proliferation and replenishes perifollicular microvasculature in mice. We construct dynamic single-cell transcriptome landscape over the course of hair regrowth and find that Que treatment stimulates the differentiation trajectory in the hair follicles and induces an angiogenic signature in dermal endothelial cells by activating HIF-1α in endothelial cells. Skin administration of a HIF-1α agonist partially recapitulates the pro-angiogenesis and hair-growing effects of Que. Together, these findings provide a molecular understanding for the efficacy of Que in hair regrowth, which underscores the translational potential of targeting the hair follicle niche as a strategy for regenerative medicine, and suggest a route of pharmacological intervention that may promote hair regrowth.

## Introduction

Hair is a uniquely mammalian trait that exerts a range of important physiological functions, including physical protection, sensory activity, thermoregulation, and social interactions (Fuchs, 2007; Xie et al., 2022). Hair loss-associated conditions such as receding hairline, diffuse hair thinning, and alopecia affect millions of people worldwide (Gilhar et al., 2012). Currently, only a few medications are clinically available for the treatment of certain subtypes of hair disorders, with some common side effects, such as hypertrichosis and local cutaneous complications (Ramos and Miot, 2015; Phillips et al., 2017). Thus, there is a vast global unmet need for a new treatment paradigm with the potential to grow hair safely and efficiently.

Hair loss is usually induced by the decline of the regenerative capability of hair follicles (HFs) that produce hair shafts and often accompanies advanced age (Matsumura et al., 2016; Ji et al., 2017). This relationship raises an intriguing question of whether compounds with potential aging-intervening effects, such as quercetin (Que) (Geng et al., 2019a; Li et al., 2021; Zou et al., 2021), metformin (Met) (Glossmann and Lutz, 2019; Soukas et al., 2019; Kulkarni et al., 2020), and gallic acid (GA) (Shan et al., 2021; Cai et al., 2022), could be used to restore HF regeneration and boost hair regrowth. Moreover, studying the cellular and molecular responses of HF to external pro-regeneration agents may offer an opportunity to reveal the principles underlying HF remodeling and facilitate the development of new strategies boosting hair growth.

Mammalian HF undergoes a precisely regulated and continuous cycle consisting of a rapid growth phase (anagen), a regression phase (catagen), and a relative quiescence or rest phase (telogen) (Morfin et al., 1979; Alonso and Fuchs, 2006; Yu et al., 2018; Simon et al., 2020; Xie et al., 2022). At the onset of anagen, hair follicle stem cells (HFSCs) become proliferative to produce and transit-amplifying cells (TACs) that produce a large amount of downstream differentiated progenies occupying the outer root sheath, companion sandwiched layer, inner root sheath (IRS), and hair shaft (HS) (Greco et al., 2009; Xu et al., 2015; Liu et al., 2021). The telogen HFs have the capability to respond rapidly to stimuli and enter anagen (Schneider et al., 2009; Cheng et al., 2018; Liu et al., 2021). Signaling pathways such as those of Wnt, Notch, and Sonic hedgehog (SHH) have been shown to play profound roles in promoting entry of HF cycles, whereas BMP signaling pathway has been reported to inhibit the transition to anagen in HFs (Fuchs, 2007; Plikus et al., 2008; Oshimori and Fuchs, 2012). Activation of HFSCs is also modulated by extrinsic microenvironmental signals supplied by vascular cells, dermal fibroblasts, sensory and sympathetic nerves, and immune cells (Hsu et al., 2014a). Particularly, branches of blood vessels arising from the deep dermal vascular plexus play a pivotal role in bringing nutrients, oxygen, and hormones to the HFs (Bassino et al., 2015; Li et al., 2019). Notably, vasculatures around HFs also go through cyclic expansion and regression synchronous with the hair growth cycle (Modlich et al., 1996). This observation suggests intimate molecular communications between skin vasculatures and HFs, although the molecular mechanisms coordinating vascular remodeling and hair regeneration remain poorly understood.

Due to the complexity of HF structure and the highly dynamic process of HF cyclic activation, it is extremely challenging to dissect pathways and cell states regulating the initiation of anagen re-entry across diverse cell types using conventional methods. The rapidly developing single-cell RNA sequencing (scRNA-seq) technology has enabled substantial progress in the characterization of cellular heterogeneity and gene expression signatures in unprecedented detail and depth (He et al., 2020; Zhang et al., 2020a; Ma et al., 2021; Fang et al., 2022; Wang et al., 2022; Xu et al., 2022; Zhou et al., 2022; Zou et al., 2022). However, scRNA-seq has not been applied to decipher the dynamics of chemical-induced hair regeneration yet.

Here, we demonstrate that administration of Que to the skin is sufficient to activate telogen HFs and initiate hair regrowth in mice. Applying scRNA-seq, we have characterized different cellular populations across multiple time points in Que-treated skin and identified alterations in cell differentiation and transcriptional networks that drive the activation of HFSCs in responding to Que. Furthermore, we have uncovered the effect of Que in upregulating the activity of HIF-1α in niche-resident endothelial cells prior to the growing phase. Based on both *in vitro* and *in vivo* evidence, we demonstrate that activation of HIF-1α promotes the proliferation and migration of endothelial cells, leading to skin vasculature remodeling and contributing to subsequent telogen-to-anagen transition of the HFs.

## Results

### Activation of hair cycles by topical application of Que

**Figure 1.**
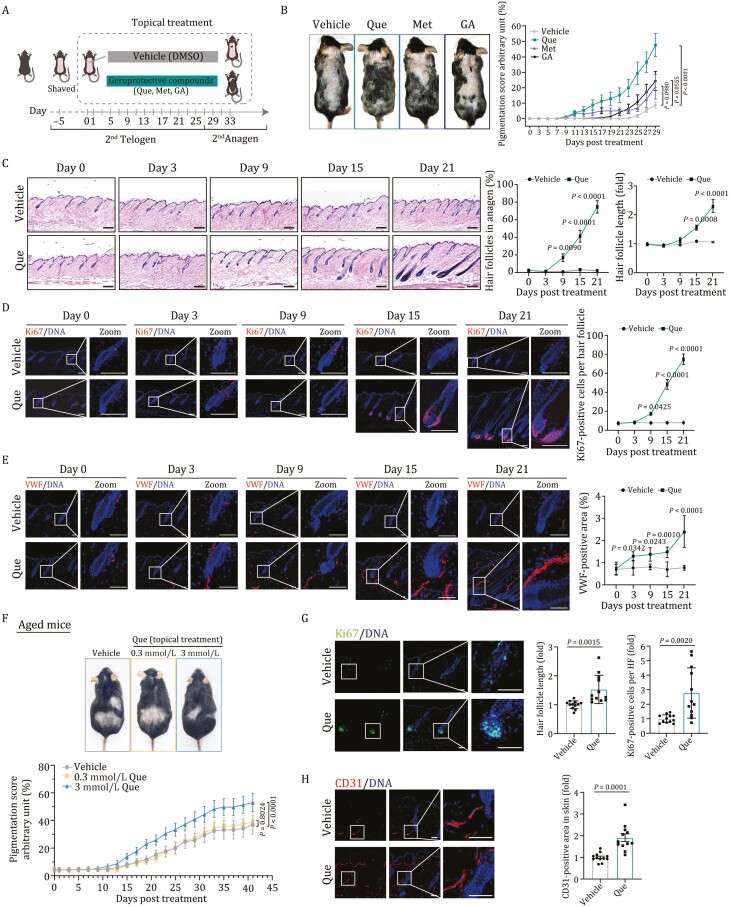
**Activation of hair cycle by topical application of Que.** (A) Schematic diagram for the time course of mouse skin treated with vehicle, Que, Met, and GA. (B) Quantification for the appearance of melanin pigmentation in mouse skin treated with vehicle, Que, Met, or GA on day 29 post-treatment. Left, representative hair coats of mice treated with vehicle, Que, Met, or GA. Right, quantification for appearance of melanin pigmentation in mouse skin treated with Que, Met, GA, or vehicle. Pigmentation scoring is described in Materials and methods. Vehicle, *n* = 20; Que, *n* = 25; Met, *n* = 8; GA, *n* = 14. Two-way ANOVA with Sidak’s test was used and data are represented as mean ± SEMs. (C) H&E staining of skin tissue sections of mice on day 0, 3, 9, 15, and 21 post-treatment with vehicle or Que. Left, representative H&E staining of skin tissue sections. Right, quantitative data of the percentage of HFs in anagen and relative HF length. Vehicle, *n* = 6; Que, *n* = 6, and 20 HFs were calculated for each individual. Two-way ANOVA with Sidak’s test was used and data are presented as the mean ± SEMs. Scale bars, 200 μm. (D) Immunostaining of Ki67 in skin tissue sections on day 0, 3, 9, 15, and 21 post-treatment with vehicle or Que. Right, quantitative data of Ki67-positive cells per HF on day 0, 3, 9, 15 and 21 post-treatment with vehicle or Que. Vehicle, *n* = 6; Que, *n* = 6, and 15 HFs were calculated for each individual. Two-way ANOVA with Sidak’s test was used and data are presented as the mean ± SEMs. Scale bars, 100 μm. (E) Immunostaining of VWF in skin tissue sections on day 0, 3, 9, 15, and 21 post-treatment with vehicle or Que. Right, quantitative data of VWF-positive area in skin on day 0, 3, 9, 15, and 21 post-treatment with vehicle or Que. Vehicle, *n* = 6; Que, *n* = 6. Two-way ANOVA with Sidak’s test was used and data are presented as the mean ± SEMs. Scale bars, 100 μm. (F) Top, representative hair coats of topical treatment with vehicle or Que (0.3 and 3 mmol/L) on 16-month mice. Bottom, quantification for appearance of melanin pigmentation in mouse skin treated with 0.3 and 3 mmol/L Que or vehicle. Vehicle, *n* = 13; 0.3 mmol/L Que, *n* = 18; 3 mmol/L Que, *n* = 18. Two-way ANOVA with Sidak’s test was used and data are presented as the mean ± SEMs. (G) Immunostaining of Ki67 in skin tissue sections on 16-month mouse skin topically treated with vehicle or 3 mmol/L Que. Right, quantitative data of Ki67-positive cells per HF in vehicle- or Que-treated groups. Vehicle, *n* = 12; Que, *n* = 12, and 15 HFs were calculated for each individual. Two-tailed unpaired Student’s *t*-test was used and data are presented as the mean ± SEMs. Scale bars, 50 μm. (H) Immunostaining of CD31 in skin tissue sections on 16-month mouse skin topically treated with vehicle or 3 mmol/L Que. Right, quantitative data of CD31-positive area in vehicle- or Que-treated groups. Vehicle, *n* = 12; Que, *n* = 12. Two-tailed unpaired Student’s *t*-test was used and data are presented as the mean ± SEMs. Scale bars, 50 μm.

We have identified several natural products capable of restoring the age-related regenerative decline of stem cells *in vitro* (Fang et al., 2018; Geng et al., 2019b; Shan et al., 2021; Zou et al., 2021). We then utilized mice with HFs synchronized into the second postnatal telogen phase, a classic model for evaluating the efficacy of regenerative strategy in promoting hair growth (Porter, 2003; Orasan et al., 2016; Son et al., 2018; Chai et al., 2019), to test the effects of these compounds *in vivo*. Que, Met, and GA were separately delivered into the shaved dorsal skin of mice for continuous treatment (Weger and Schlake, 2005; Chai et al., 2019) (Fig. 1A). Because follicular melanogenesis is strictly coupled with HF cycling, we recorded the macroscopically visible changes in skin pigmentation during 29 days of treatment to assess the degree of hair regrowth (Fig. 1B). Consistent with the previous report on the duration of physiological telogen (Schneider et al., 2009), scattered pigment spots in the vehicle-treated group were not observed until at day 25 of treatment (Fig. 1B). In contrast, mice treated with Que, Met, and GA all exhibited accelerated skin pigmentation, starting at day 9, 15, and 19, respectively (Fig. 1B). Further titration of Que from 0.1 to 30 mmol/L showed the dose of 0.3 mmol/L resulted in the best effect in anagen induction (Fig. S1A).

Over the course of Que-induced HF regeneration, histological analysis demonstrated that the proportion of HFs in the anagen phase and the average length of HFs increased continuously (Fig. 1C). Consistent with the anagen re-entry, Ki67-positive proliferating cells progressively accumulated in the matrix at the HF base (Fig. 1D). Compared to vehicle treatment, Que-treated mice showed increased vasculatures, as marked by von Willebrand factor (VWF) immunostaining, underneath the hair germ, especially in the perifollicular regions (Fig. 1E). Moreover, oral gavage of Que in mice at the second postnatal telogen phase for 2 months also obviously stimulated hair regrowth and enhanced skin vascularization (Fig. S1B–D). In addition, topical application of Que on the skin of 16-month mice for 43 days and oral gavage of Que in aged mice for 8 months partially rescued the age-related hair loss and enhanced skin vascularization (Figs. 1F–H, S1E and S1F). Collectively, our data provided evidence that Que treatment promoted hair regeneration and anagen-associated angiogenesis.

### Single-cell transcriptomic profiling of the skin treated with Que

**Figure 2.**
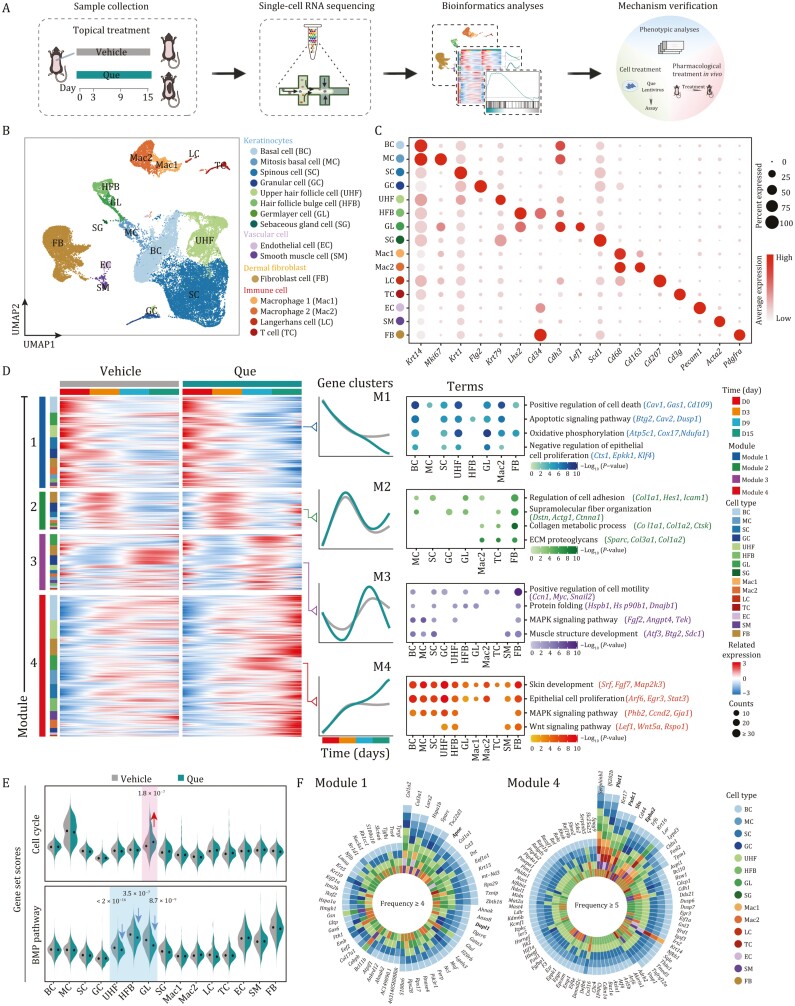
**Single-cell transcriptional profile analyses of different cell types with Que treatment**. (A) Workflow showing the procedure of scRNA-seq, phenotypic analysis, and mechanism verification of hair growth activated by topical application of Que. (B) UMAP plot showing the fifteen cell types in mouse skin. Cells types are annotated on the right. BC, basal cell; MC, mitotic basal cell; SC, spinous cell; GC, granular cell; UHF, upper HF cell; HFB, HF bulge cell; GL, germ layer cell; SG, sebaceous gland cell; EC, endothelial cell; SM, smooth muscle cell; FB, fibroblast cell; Mac1, Macrophage 1; Mac2, Macrophage 2; LC, Langerhans cell; TC, T cells. (C) Dot plots showing the expression of representative genes for each cell type in skin. (D) Heatmaps showing the expression profiles of differentially expressed genes along the time trajectory (TDEGs) for different cell types in vehicle- and Que-treated groups, which were divided into four clusters based on the expression pattern. Representative GO terms and pathways of TDEGs in the corresponding clusters are shown on the right. (E) Violin plots showing gene set scores of cell cycle and BMP pathway in different cell types of vehicle- and Que-treated groups. (F) Plots showing the continuously downregulated TDEGs (left, module 1) shared by at least four cell types and consistently upregulated TDEGs (right, module 4) shared by at least five cell types.

To reveal the cellular and molecular dynamics of chemical-induced hair regrowth, we collected the mouse skin with Que or vehicle treatment at four post-treatment time points: day 0 (base line before treatment), day 3 (priming response period), day 9 (pigment detected), and day 15 (anagen HFs accumulated) (Fig. 2A). The scRNA-seq data from a total of 35,358 cells that met quality control metrics were applied to unsupervised clustering via uniform manifold approximation and projection (UMAP) to resolve cell-type composition at each time point (Fig. S2A–F).

Based on the known cell type-specific markers and transcriptional features, we identified 15 cell types that can be grouped into four main cellular categories, including keratinocytes, vascular cells, fibroblasts, and immune cells (Figs. 2B and S2G). As expected, keratinocytes, which were present in both epidermis and HFs, made up the largest cell population in the skin. The epidermal keratinocytes included basal cell (BC, *Krt14*^+^), mitotic basal cell (MC, *Mki67*^+^), spinous cell (SC, *Krt1*^+^), and granular cell (GC, *Flg2*^+^), whereas keratinocytes from HFs included upper HF cell (UHF, *Krt79*^+^), HF bulge cell (HFB, *Lhx2*^*+*^, and *Cd34*^+^), germlayer cell (GL, *Cdh3*^+^, and *Lef1*^+^), and sebaceous gland cell (SG, *Scd1*^+^) (Fig. 2B and 2C). Moreover, TACs, a group of early intermediate in hair regeneration originated from HFSCs with highly expressed *Lef1* and *Mki67* (Hsu et al., 2014b), were contained in GL (Figs. 2B and S2H). The other three categories of cells that resided in HF microenvironment included: (i) vascular cells such as endothelial cell (EC, *Pecam1*^+^) and smooth muscle cell (SM, *Acta2*^+^), (ii) fibroblast cell (FB, *Pdgfra*^+^), and (iii) various immune cells such as Macrophage 1 (Mac1, *Cd68*^+^), Macrophage 2 (Mac2, *Cd163*^+^), Langerhans cell (LC, *Cd207*^+^), and T cell (TC, *Cd3g*^+^) (Fig. 2B and 2C; Table S1). Notably, although cell identities did not show any substantial shift, the proportion of HF keratinocytes expanded dramatically after Que treatment, consistent with the enhanced hair growth (Figs. 1C, 1D and S2E–F). Overall, our analyses delineates the cellular landscape of HFs and their surrounding niches during Que-induced regeneration at the single-cell resolution.

### A time-resolved model dissecting molecular transition during Que-induced hair growth

To infer transcriptional dynamics of Que-induced hair growth, we performed a time-ordering analysis over the course of hair regeneration (Fig. 2D). Differentially expressed genes along the time trajectory (TDEGs) were identified and classified into four modules based on their transcriptional kinetics (Fig. 2D; Tables S2 and S3). Module 1 comprised TDEGs whose expression continuously decreased since hair clipping in both Que- and vehicle-treated skin samples but decreased more with Que treatment, especially in BC, and HF-related cell types UHF and GL (Figs. 2D and S2I). Gene Ontology (GO) analysis showed that module 1 genes were enriched in positive regulation of cell death (*Btg2*, *Cav2*, and *Dusp1*) and oxidative phosphorylation (*Atp5c1*, *Cox17*, and *Ndufa1*) (Fig. 2D; Table S3), consistent with the previous discovery that the inhibition of mitochondrial oxidative phosphorylation stimulated the anagen phase and accelerated HF regeneration (Kondo et al., 2002). Module 2 contained TDEGs with a bell-shaped expression kinetics, the expression of these genes reached the peak at intermediate time points and decreased at later time points, and the extent of this decrease was slightly enhanced by the Que treatment (Fig. 2D). These TDEGs were associated with cell adhesion (*Col1a1*, *Hes1*, and *Icam1*) and regulation of collagen metabolic process (*Col1a1*, *Col1a2*, and *Ctsk*) and were mostly attributed to FB, indicating the influence of extracellular matrix (ECM) remodeling on early HF development (Figs. 2D and S2I; Table S3). We also identified a set of TDEGs with cosine-shaped expression kinetics (module 3), their expression decreased sharply initially, increased subsequently, and recovered at later time points. The degree of these changes was enhanced by the Que treatment (Fig. 2D). These genes were enriched in GO terms of positive regulation of cell motility (*Ccn1*, *Myc,* and *Snail2*) and MAPK signaling pathway (*Fgf2*, *Angpt4*, and *Tek*), especially in GL, FB, and GC and might be related to the escape from the quiescent state at the early phase of telogen–anagen transition (Su et al., 2020) (Figs. 2D and S2I; Table S3). Module 4 contained about 43% of total TDEGs, and the expression of module 4 genes increased gradually upon Que treatment but almost plateaued in the vehicle-treated group after day 3 (Fig. 2D). These genes were mainly enriched in epidermal cells and HF cells, such as BC, UHF, and GL, and widely involved in epithelial cell differentiation (*Sfn*, *Abl2*, and *Dll1*), the MAPK signaling pathway (*Phb2*, *Ccnd2*, and *Gja1*), and the Wnt signaling pathway (*Wnt5a*, *Lef1*, and *Rspo1*) that reported to be associated with anagen onset (Figs. 2D and S2I; Table S3). In addition, we also revealed that Que treatment repressed BMP signaling pathway (Fig. 2E; Table S4), which was demonstrated previously to inhibit hair growth (Oshimori and Fuchs, 2012; Foitzik et al., 2000; Joost et al., 2020). Moreover, scores for cell cycle-related genes were increased in HF cells, especially in GL, upon Que treatment, in line with increased proliferative cells (Fig. 2E; Table S4). Altogether, our data delineate the temporal dynamics of cell type-specific molecular events underlying Que-induced hair regeneration.

To identify the key genes involved in Que-initiated hair growth, we further investigated the high-frequency TDEGs shared across multiple cell types in the four modules (Figs. 2F and S2J). We identified 90 genes consistently upregulated (module 4) in at least five cell types and 63 genes consistently downregulated (module 1) in at least four cell types (Fig. 2F). High-frequency upregulated TDEGs included *Plet1* and *Sfn* that were highly correlated with epithelial cell differentiation (Kondo et al., 2002; Hammond et al., 2012; Gunnarsson et al., 2016) and vasculature development-related genes *Pxdc1* and *Epha2* (Tian et al., 2020; Zhang et al., 2021a). The consistently downregulated TDEGs *Apoe* and *Dapl1* were both reported to be involved in the positive regulation of cell death (Kobayashi and Yonehara, 2009; Medkour et al., 2017; Martins Cardoso et al., 2019) (Fig. 2F). Taken together, our data identify transcriptomic signatures and key genes for the hair growth stimulated by the Que treatment.

### Hierarchical HFSC differentiation is activated upon Que treatment

**Figure 3.**
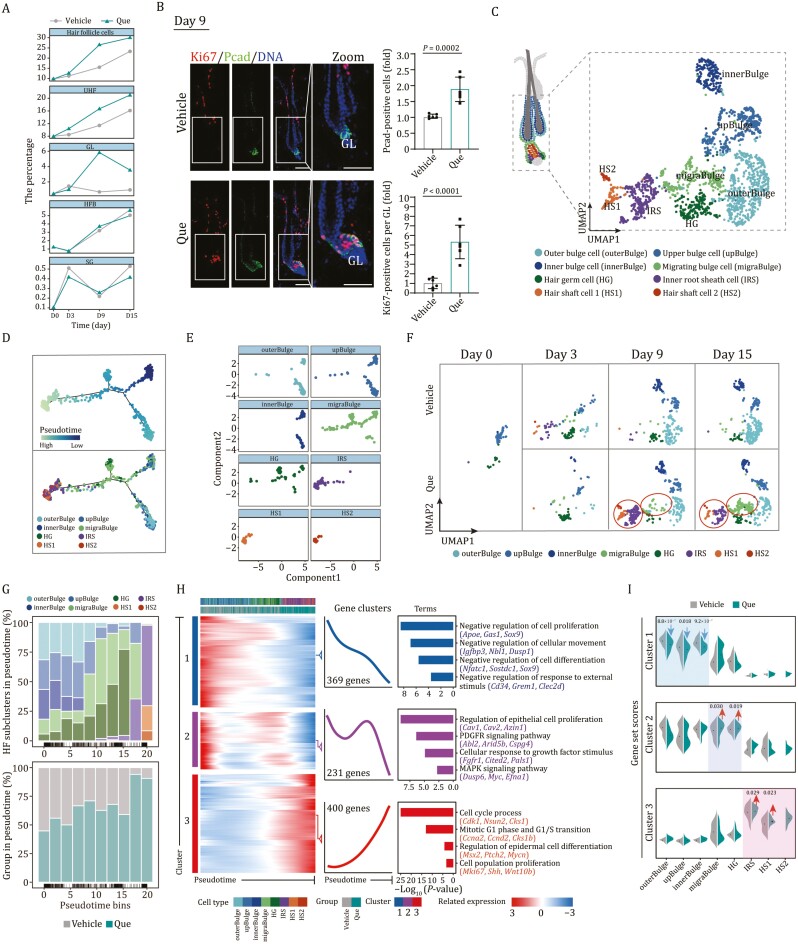
**Single-cell transcriptomic changes of HF subclusters upon Que treatment**. (A) The line plots showing the proportions of HF cells and HF subgroups in skin tissues after treatment with vehicle or Que at different time points. (B) Immunofluorescence staining of Ki67 and GL marker P-cadherin (Pcad) in skin tissues on day 9 post-treatment with vehicle or Que. Right, the quantitative data of GL and Ki67-positive cells in GL. GL, germ layer cells. Vehicle, *n* = 6; Que, *n* = 6, and 15 HFs were calculated for each individual. Two-tailed unpaired Student’s *t*-test was used and data are presented as the mean ± SEMs. Scale bars, 50 μm. (C) UMAP plot showing the distribution of cells in the cycling part of HFs. HF sub-cell types are annotated on the bottom. outerBulge, outer bulge cell; upBulge, upper bulge cell; innerBulge, inner bulge cell; migraBulge, migrating bulge cell; HG, hair germ cell; IRS, IRS cell; HS1, hair shaft cell 1; HS2, hair shaft cell 2. (D) Pseudotime trajectory analysis of cells in cycling HFs. Inferred pseudotime for each cell type is shown on the top. The cell types along the pseudotime trajectories are shown on the bottom. (E) The plots showing the distribution of HF subclusters along with the pseudotime trajectory. (F) UMAP plots showing the distribution of different HF cells in the cycling HFs on indicated days. (G) Binned pseudotime analyses displaying HF subclusters ordered on the pseudotime axis. Bars are shown with different sub-cell types (top) and groups (bottom). (H) Heatmaps showing the expression profiles of genes along with the pseudotime of different HF subclusters vehicle- and Que-treated groups, which were divided into three clusters with the expression pattern. The enriched GO terms for the corresponding cluster were listed on the right. (I) Violin plots showing the AUC scores of gene set corresponding to clusters in different HF subclusters of vehicle- and Que-treated groups.

HFs are complex mini-organs responsible for the generation of HSs. The well-organized structure of HFs is orchestrated by a panel of heterogeneous stem cells with lineage hierarchy and rapidly dividing cells surrounding the dermal papilla located at the base bulb of a HF (Greco et al., 2009). Focusing on HFs, we found that proportions of HF keratinocytes increased as the activation of the hair growth upon stimulation by Que (Fig. 3A). In particular, our immunostaining results confirmed that the fraction of proliferating cells in the hair germ, where the matrix cells divide to comprise the HS, was five times higher in the Que-treated group than that in the vehicle-treated group, contributing to an enlarged germlayer cell population (Fig. 3B).

To reveal the heterogeneity and dynamics of HFSCs in response to Que treatment, we further classified cycling HF cells (including HFB and GL) into eight subsets (Figs. 3C and S3A; Table S1) and used the lineage trajectory analysis to infer the prospective differentiation path of these subpopulations (Fig. 3C and 3E). The subpopulations that displayed sequentially along the differentiation trajectory could be mapped to different bulge regions (outer bulge cell, outerBulge, *Krt24*^+^; inner bulge cell, innerBulge, *Fgf18*^+^; upper bulge cell, upBulge, *Pthlh*^+^), all of which highly expressed the classic HFSC markers *Nfatc1*, *Sox9*, and *Foxc1* (Fig. S3B). In addition, we defined a distinct subset of migrating bulge cell (migraBulge, *Bgn*^+^) that presented a decreasing quiescence score based on the overall expression level of target genes of *Foxc1*, a key transcription factor reinforcing quiescence of HFSCs (Wang et al., 2016; Chovatiya et al., 2021), compared to that of innerBulge (Fig. S3A and S3C; Tables S1 and S4). In addition to bulge cells, HF cells derived from GL were classified into four subpopulations, including (i) hair germ cell (HG, *Icam1*^+^), a small cell cluster between the bulge and dermal papilla derived from bulge stem cell and responsible for the initiation of cyclic hair regeneration; (ii) inner root sheath cell (IRS, *Krt25*^+^); (iii) hair shaft cell 1 (HS1, *Fabp4*^+^), and (iv) hair shaft cell 2 (HS2, *Krt35*^+^) (Figs. S3A and 3C–E; Table S1). The latter three types of cells are mature cells that envelop the differentiation core of HF. Notably, being characteristics of newborn HF, migraBulge, and HG distributed along the cell differentiation trajectory, which confirmed that the dynamic HF subpopulations during the telogen–anagen transition were captured in our analysis (Fig. 3D and 3E).

We then compared the dynamics of cellular composition between vehicle- and Que-treated groups. Despite minimal cell composition changes were observed on day 3, the migraBulge population emerged on day 9 and continuously expanded on day 15 post-Que treatment (Fig. 3F and 3G). In contrast, the migraBulge population was almost absent during the entire process in the vehicle-treated group without obvious hair growth, suggesting that the migraBulge population is specialized for HF regeneration (Fig. 3F and 3G). Similarly, IRS and HS subpopulations were exclusively observed in the Que-treated group since day 9 post-treatment (Fig. 3F and 3G). Thus, HFSCs, which were normally maintained in the undifferentiated states, were activated by the Que treatment to proliferate and differentiate, and thereby governed the entry of the anagen phase.

To elaborate on how anagen transition was affected by the Que treatment, we delineated cell type-specific gene expression profiles along the differentiation trajectory (Fig. 3H). Based on the top 1000 DEGs along with the pseudotime differentiation wave (wave-DEGs), we defined 3 distinct gene clusters and depicted successive waves of gene expression (Fig. 3H). Cluster 1 of wave-DEGs was highly expressed in slow-cycling bulge stem cells and progressively downregulated along the trajectory (Fig. 3H). Accordingly, cluster 1 wave-DEGs were involved in the repression of cell proliferation and were enriched with *NfatC1*, *Foxc1*, and *Sox9* encoding transcription factors that maintained the quiescent state of HFSCs (Horsley et al., 2008; Kadaja et al., 2014; Wang et al., 2016) (Figs. 3H, 3I, and S3D). Consistent with their general roles in the morphogenesis of epithelium, cluster 2 wave-DEGs were expressed relatively low at the beginning, higher in the intermediate progenitors responding to growth stimulation, and sharply decreased at the end of cell differentiation, suggesting that their expressions tracked with the re-entry of anagen (Fig. 3H and 3I). Cluster 3 wave-DEGs were progressively upregulated along the differentiation trajectory, highly expressed in hair germ-derived cell types, and were involved in the regulation of the cell cycle process and epidermal development (Fig. 3H and 3I). Accordingly, *Lef1*, *Msx2*, and *Hoxc13*, encoding key transcription factors dictating hair matrix cell differentiation (DasGupta and Fuchs, 1999; Jave-Suarez et al., 2002; Wu et al., 2010; Kim and Yoon, 2013; Joost et al., 2020), were specifically enriched in cluster 3 (Fig. S3D). Moreover, we observed prominent increases in the activity of Wnt and SHH signalings in migraBulge and decrease of BMP signaling in bulge cells and HG upon Que treatment (Fig. S3E; Table S4). Collectively, these data revealed that the exposure of Que rewired the continuum of HFSC differentiation and boosted HF regeneration.

### Rapid vascularization in response to Que treatment

**Figure 4.**
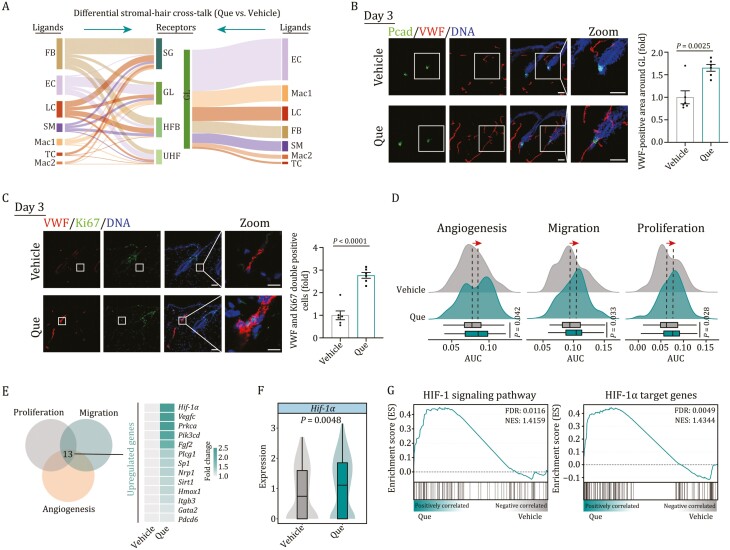
**Vascular endothelial cells undergo remodeling with activated HIF-1α signaling after Que treatment**. (A) Sankey plots showing changes (Que versus vehicle) in the number of potential receptor-ligand interaction events between HF subclusters and other niche cell types. Cell types were ordered by the changes of receptor-ligand pair interaction strength. (B) Immunofluorescence staining of endothelial cell marker VWF and GL marker P-cadherin (Pcad) in skin tissues on day 3 post-treatment with vehicle or Que. Right, the quantitative data of VWF-positive area around GL. Vehicle, *n* = 6; Que, *n* = 6, and 15 GLs were calculated for each individual. Two-tailed unpaired Student’s *t*-test was used and data are presented as the mean ± SEMs. Scale bars, 50 μm. (C) Immunofluorescence staining of VWF and Ki67 in skin tissue on day 3 post-treatment with vehicle or Que. Right, the quantitative data of Ki67-positive cells in VWF-positive cells. Vehicle, *n* = 6; Que, *n* = 6, and 15 HFs were calculated for each individual. Two-tailed unpaired Student’s *t*-test was used and data are presented as the mean ± SEMs. Scale bars, 50 μm. (D) Ridge plots showing AUC scores of gene set related to angiogenesis, migration, and proliferation in endothelial cells on day 3 post-treatment with vehicle or Que. (E) Venn plot showing the number of genes shared among gene sets related to angiogenesis, migration, and proliferation. The expression profiles of shared genes were shown on the right. (F) Violin and box plot showing the transcriptional expression level of *Hif-1*α in endothelial cells on day 3 post-treatment with vehicle or Que. (G) Gene set enrichment analysis (GSEA) showing HIF-1 signaling pathway (left) and HIF-1α target genes (right) enriched in endothelial cells on day 3 post-treatment with vehicle or Que. NES, normalized enrichment score; FDR, false discovery rate.

The cyclic regeneration of hair responds to the complex cellular interactions between stem cells and their surrounding niches where HF resides and organizes into an intricate structure (Fuchs, 2007). To study whether Que resets the stem cell niche to a favorable environment for HF growth, we performed a receptor-ligand analysis between cycling HF subpopulations and major niche cells, including FB, EC, LC, SM, Mac1, TC, and Mac2, and found that the interactions between HF subpopulations and stromal cells were augmented by Que treatment (Fig. 4A). In particular, we predicted a number of potential receptor-ligand communications between GL and EC (Fig. 4A), most of which were *Fabp4* and *Aqp1-*positive blood vascular endothelial cells (Fig. S4A and S4B). Detailed inspection revealed that Que treatment strengthened the GL–EC interaction mediated through Wnt and Notch pathways, which had been reported to initiate the HF anagen entry previously (Greco et al., 2009; Hsu and Fuchs, 2012; Bassino et al., 2015) (Fig. S4C). Furthermore, our data showed that Que treatment induced the early onset of endothelial proliferation and angiogenesis concurrent with the entry of anagen (Figs. 1E, 4B, 4C and S4D), which collectively suggested that the cross-talk between the skin vasculature and HF contributed to Que-induced hair regrowth.

To further validate the early-onset pro-angiogenic effects of Que, we assessed the activity of gene sets related to angiogenesis, migration, and proliferation, and found the scores of all these gene sets were increased to be upregulated at day 3 post-Que treatment (Fig. 4D). Notably, the expressions of *Hif-1α* and its target gene *Sirt1* were higher in endothelial cells from Que-treated skin compared with vehicle-treated counterpart (Figs. 4E, 4F, S4E and S4F). In addition, GSEA analysis showed that the HIF-1 signaling pathway and HIF-1α target genes were upregulated by Que treatment in vascular endothelial cells (Fig. 4G), and an increased protein level of HIF-1α was observed in skin tissues upon Que treatment (Fig. S4G). Collectively, our results indicate that vascular endothelial cells undergo remodeling with activated HIF-1 pathway and potentially strengthened interaction with growing HF cells.

### Que-induced dermal angiogenesis via the activation of the HIF-1α pathway

**Figure 5.**
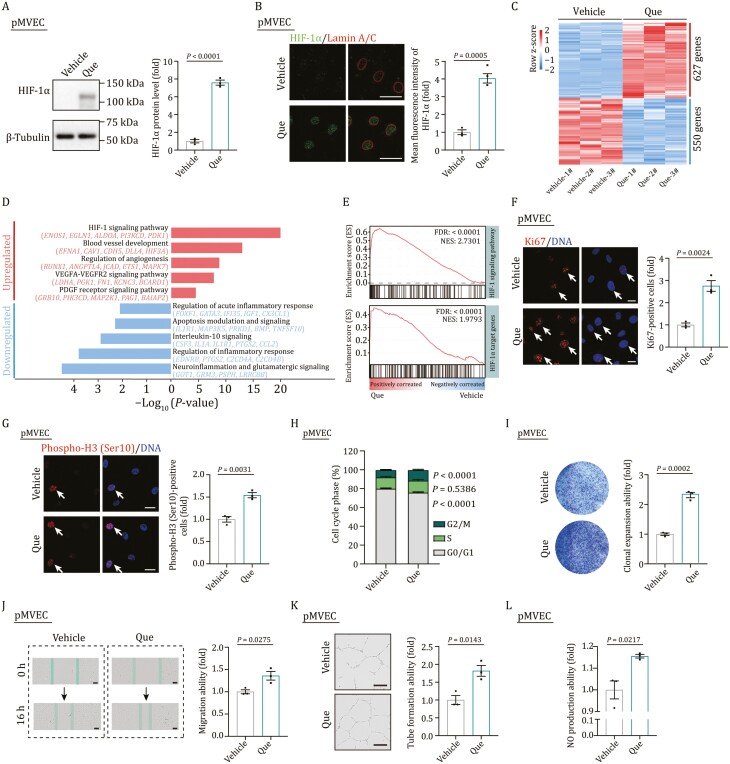
**Que treatment promotes the migration and proliferation of endothelial cells via the activation of HIF-1α**. (A) Western blot analysis of HIF-1α in pMVECs treated with vehicle or Que. *n* = 3 biological replicates. Two-tailed unpaired Student’s *t*-test was used and data are presented as the mean ± SEMs. Scale bars, 50 μm. (B) Immunostaining of HIF-1α and Lamin A/C in pMVECs treated with vehicle or Que. Right, the quantitative data of mean fluorescence intensity of HIF-1α in the nucleus. *n* = 3 biological replicates. Two-tailed unpaired Student’s *t*-test was used and data are presented as the mean ± SEMs. Scale bars, 50 μm. (C) Heatmap showing the relative expression level (row-scaling) of DEGs in pMVECs treated with Que versus vehicle. (D) Representative GO terms and pathways of upregulated and downregulated DEGs in pMVECs treated with Que versus vehicle. (E) GSEA showing HIF-1 signaling pathway (top) and HIF-1α target genes (bottom) enriched in pMVEC treated with vehicle or Que. NES, normalized enrichment score; FDR, false discovery rate. (F) Immunostaining of Ki67 in vehicle or Que-treated pMVECs. Right, the quantification of Ki67-positive cells in vehicle or Que-treated pMVECs. *n* = 3 biological replicates. Two-tailed unpaired Student’s *t*-test was used and data are presented as the mean ± SEMs. Scale bars, 20 μm. Arrows indicate Ki67-positive cells. (G) Immunofluorescence staining of Phospho-H3 (Ser10) in pMVECs treated with vehicle or Que. Right, the quantification of Phospho-H3 (Ser10)-positive cells in vehicle or Que-treated pMVECs. *n* = 3 biological replicates. Two-tailed unpaired Student’s *t*-test was used and data are presented as the mean ± SEMs. Scale bars, 25 μm. Arrows indicate Phospho-H3 (Ser10)-positive cells. (H) Cell cycle analysis of pMVECs treated with vehicle or Que. *n* = 3 biological replicates. Two-way ANOVA with Sidak’s test was used and data are represented as mean ± SEMs. (I) Clonal expansion analysis of pMVECs treated with vehicle or Que. *n* = 3 biological replicates. Two-tailed unpaired Student’s *t*-test was used and data are presented as the mean ± SEMs. (J) Wound scratch assay for detecting the migration of pMVECs treated with vehicle or Que. *n* = 3 biological replicates. Two-tailed unpaired Student’s *t*-test is used and data are presented as the mean ± SEMs. Scale bars, 100 μm. (K) Angiogenesis was assessed by the formation of capillary-like tubes of pMVECs treated with vehicle or Que *in vitro*. *n* = 3 biological replicates. Two-tailed unpaired Student’s *t*-test was used and data are presented as the mean ± SEMs. Scale bars, 100 μm. (L) NO production ability of pMVECs treated with vehicle or Que by FACS analysis. *n* = 3 biological replicates. Two-tailed unpaired Student’s *t*-test was used and data are presented as the mean ± SEMs. Scale bars, 50 μm.

To unravel the molecular mechanism underlying the angiogenesis stimulated by Que, we treated the primary skin microvascular endothelial cells (pMVECs) with 100 nmol/L Que. In agreement with our observation *in vivo*, Western blot analysis showed that the protein level of HIF-1α was elevated by Que treatment in pMVECs (Fig. 5A). Immunostaining further confirmed that Que treatment increased the protein level of HIF-1α as well as the active form of nuclear trans-localized HIF-1α in pMVECs (Fig. 5B). Whole transcriptome profiling and GSEA showed that the upregulated genes in Que- versus vehicle-treated pMVECs were mainly enriched in the HIF-1 signaling pathway, blood vessel development, and regulation of angiogenesis (Fig. 5C–E; Table S5). More importantly, we found that Que treatment promoted proliferation, migration, tube formation, and nitric oxide (NO) production (an indicator of activation of endothelial cells) in pMVECs (Fig. 5F–L), all of which were consistent with vascular replenishment stimulated by Que *in vivo*. Thus, these findings suggest that Que stimulates the activation of HIF-1 signaling in endothelial cells and promotes angiogenesis both *in vitro* and *in vivo*.

**Figure 6.**
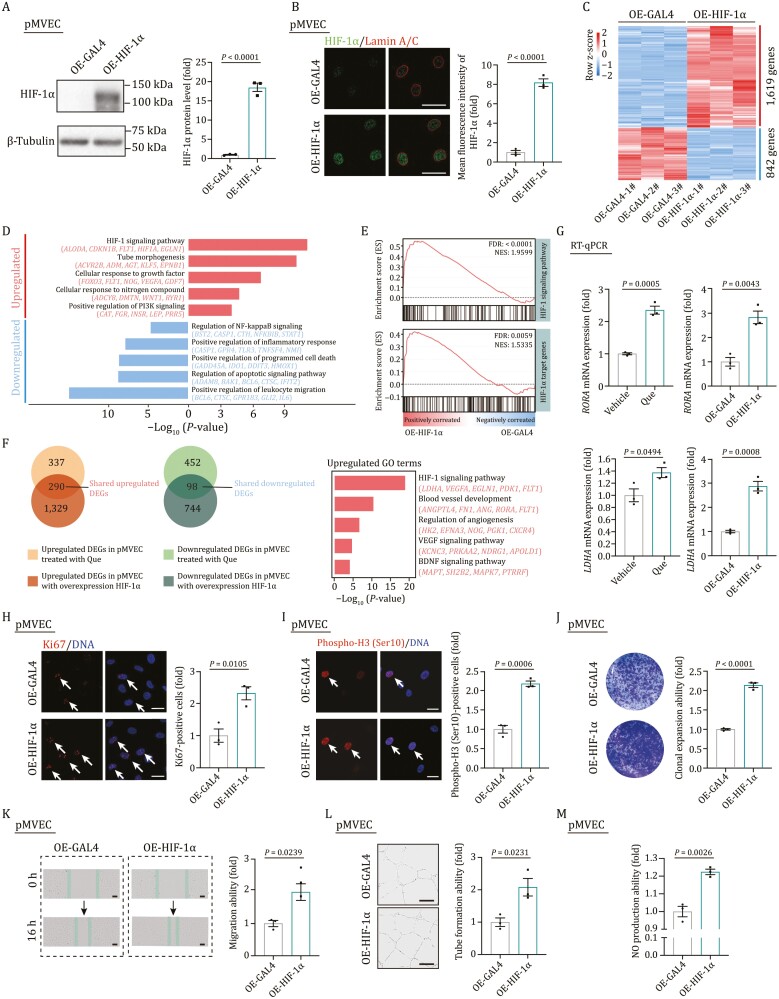
**Overexpression of HIF-1α promotes the migration and proliferation of pMVECs**. (A) Western blot analysis of HIF-1α in pMVEC with overexpressing GAL4 or HIF-1α. OE-GAL4, overexpressing GAL4; OE-HIF-1α, overexpressing HIF-1α. *n* = 3 biological replicates. Two-tailed unpaired Student’s *t*-test was used and data are presented as the mean ± SEMs. (B) Immunostaining of HIF-1α and Lamin A/C in pMVECs with overexpressing GAL4 or HIF-1α. Right, the quantitative data of mean fluorescence intensity of HIF-1α in the nucleus. *n* = 3 biological replicates. Two-tailed unpaired Student’s *t*-test was used and data are presented as the mean ± SEMs. Scale bars, 50 μm. (C) Heatmap showing the relative expression level (row-scaling) of DEGs in pMVECs with overexpressing HIF-1α versus GAL4. (D) Representative GO terms and pathways of upregulated and downregulated DEGs in pMVECs with overexpressing HIF-1α versus GAL4. (E) GSEA showing HIF-1 signaling pathway (top) and HIF-1α target genes (bottom) enriched in pMVECs with overexpressing GAL4 or HIF-1α. NES, normalized enrichment score; FDR, false discovery rate. (F) Venn plots showing the numbers of shared upregulated (left) and downregulated (right) DEGs, and representative GO terms and pathways of shared upregulated DEGs in pMVECs treated with Que and overexpressing HIF-1α. (G) RT-qPCR analysis of shared upregulated genes *RORA* and *LDHA* in pMVECs treated with Que or overexpressing HIF-1α. *n* = 3 biological replicates. Two-tailed unpaired Student’s *t*-test was used and data are presented as the mean ± SEMs. (H) Immunostaining of Ki67 and quantification of Ki67-positive cells in pMVECs with overexpressing GAL4 or HIF-1α. *n* = 3 biological replicates. Two-tailed unpaired Student’s *t*-test was used and data are presented as the mean ± SEMs. Scale bars, 20 μm. Arrows indicate Ki67-positive cells. (I) Immunofluorescence staining of Phospho-H3 (Ser10) and the quantification of Phospho-H3 (Ser10)-positive cells in pMVECs with overexpressing GAL4 or HIF-1α. *n* = 3 biological replicates. Two-tailed unpaired Student’s *t*-test was used and data are presented as the mean ± SEMs. Scale bars, 25 μm. Arrows indicate Phospho-H3 (Ser10)-positive cells. (J) Clonal expansion analysis of pMVECs with overexpressing GAL4 or HIF-1α. *n* = 3 biological replicates. Two-tailed unpaired Student’s *t*-test was used and data are presented as the mean ± SEMs. (K) Wound scratch assay for detecting the migration ability of pMVECs with overexpressing GAL4 or HIF-1α. *n* = 3 biological replicates. Two-tailed unpaired Student’s *t*-test was used and data are presented as the mean ± SEMs. Scale bars, 100 μm. (L) Angiogenesis was assessed by the formation of capillary-like tubes of pMVECs with overexpressing GAL4 or HIF-1α. *n* = 3 biological replicates. Two-tailed unpaired Student’s *t*-test was used and data are presented as the mean ± SEMs.Scale bars, 100 μm. (M) NO production ability of pMVECs with overexpressing GAL4 or HIF-1α by FACS analysis. *n* = 3 biological replicates. Two-tailed unpaired Student’s *t*-test was used and data are presented as the mean ± SEMs.

To further validate the causal role of HIF-1α in angiogenesis, we overexpressed HIF-1α in pMVECs (Fig. 6A and 6B). Notably, about half of genes responding to HIF-1α overexpression overlapped with genes responding to Que treatment, and these overlapped genes are primarily associated with endothelial cell proliferation (Fig. 6C–F; Table S5). Among these overlapped genes, the upregulation of two HIF-1α downstream genes *RORA* and *LDHA* were validated in both conditions via RT-qPCR (Fig. 6G). More importantly, HIF-1α overexpression largely reproduced the phenotypes we observed in Que-treated cells, including the increased proliferation, migration, tube formation, and NO production (Fig. 6H–M). Overall, our data imply the pivotal roles of HIF-1α in Que-triggered angiogenesis.

### The HIF-1α agonist DMOG induces hair growth *in vivo*

**Figure 7.**
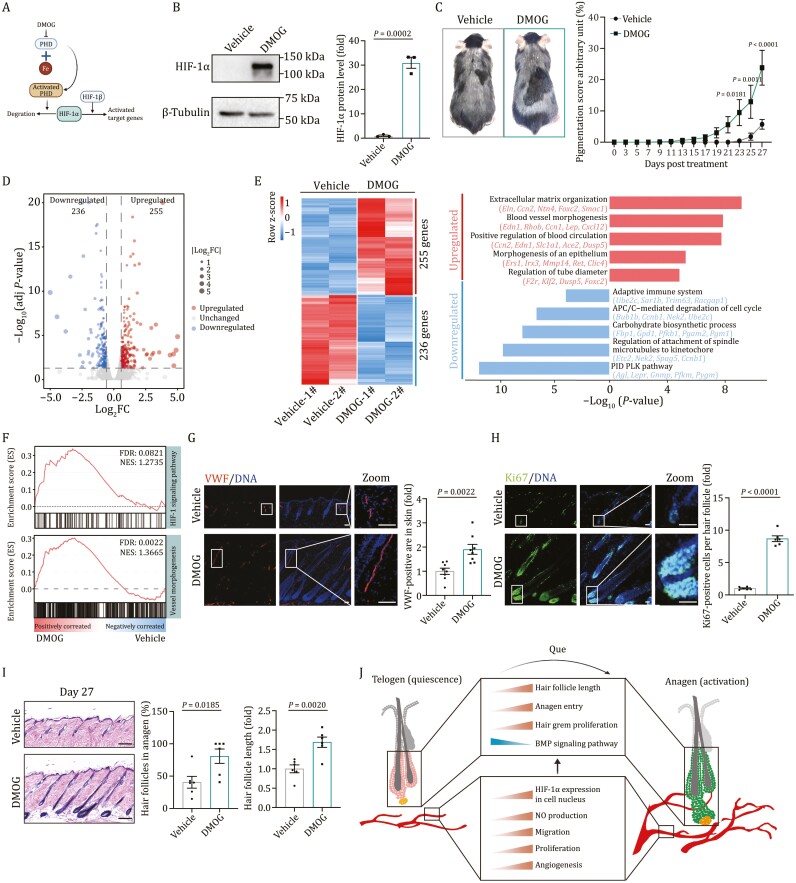
**HIF-1**α **activator DMOG induces hair growth *in vivo***. (A) The schematic illustration showing DMOG increased HIF-1α stabilization and activated the target genes through inhibiting Prolyl hydroxylase (PHD). (B) Western blot analysis of HIF-1 α in pMVECs treated with DMOG. *n* = 3 biological replicates. Two-tailed unpaired Student’s *t*-test was used and data are presented as the mean ± SEMs. (C) Left, representative hair coats of vehicle- or DMOG-treated mice on day 27 post-treatment. Right, quantification for the appearance of melanin pigmentation in mouse skin treated with DMOG versus vehicle. Pigmentation scoring is described in Materials and methods. Vehicle, *n* = 14; DMOG, *n* = 17. Two-tailed unpaired Student’s *t*-test was used and data are presented as the mean ± SEMs. (D) Volcano plot showing the expression patterns DEGs of mouse skin treated with DMOG versus vehicle. (E) Heatmap showing the relative expression level (row-scaling) of DEGs (left) and representative GO terms and pathways (right) for upregulated and downregulated DEGs in mouse skin treated with DMOG versus vehicle. (F) GSEA showing HIF-1 signaling pathway (top) and blood vessel morphogenesis (bottom) enriched in mouse skin treated with vehicle or DMOG. NES, normalized enrichment score; FDR, false discovery rate. (G) Immunofluorescence staining of VWF in skin tissues on day 27 post-treatment with vehicle or DMOG. Right, the quantitative data of VWF-positive area in the skin. Vehicle, *n* = 8; DMOG, *n* = 8. Two-tailed unpaired Student’s *t*-test was used and data are presented as the mean ± SEMs. Scale bar, 100 μm. (H) Immunofluorescence staining of Ki67 in HFs on day 27 post-treatment with vehicle or DMOG. Right, the quantitative data of Ki67-positive cells per HF. Vehicle, *n* = 6; DMOG, *n* = 6 and 15 HFs were calculated for each individual. Two-tailed unpaired Student’s *t*-test was used and data are presented as the mean ± SEMs. Scale bars, 50 μm. (I) H&E staining of skin tissues sections on day 27 post-treatment with vehicle or DMOG (left) and the quantitative data of the percentage of anagen HFs and the relative HF length in skin tissue (right). Vehicle, *n* = 6; DMOG, *n* = 6. Two-tailed unpaired Student’s *t*-test was used and data are presented as the mean ± SEMs. Scale bars, 200 μm. (J) A schematic illustration showing the phenotypical and molecular changes during the hair regrowth upon Que treatment.

To determine whether activation of HIF-1 signaling is sufficient to induce hair growth *in vivo*, we topically applied dimethyloxallyl glycine (DMOG), of which the HIF-1α activation effects have been well validated previously (Yuan et al., 2014; Jahangir et al., 2018; Shi et al., 2021) and in this study (Fig. 7A and 7B), to mouse models with a procedure similar to the Que treatment. We noticed the accelerated skin pigmentation *in vivo* upon DMOG treatment (Fig. 7C). The whole-transcriptome analysis demonstrated that the treatment of DMOG, similar to Que, activated HIF-1 signaling pathway, as well as increased the expression of genes involved in blood vessel development (Fig. 7D–7F; Table S5). Consistently, we found increased protein level of HIF-1α in skin tissues upon DMOG treatment (Fig. S4H). In addition, DMOG induced angiogenesis as evidenced by an increase in VWF-positive endothelial cells (Fig. 7G). Importantly, we found that DMOG treatment increased the percentage of proliferating cells in HFs, the proportion of HFs in the anagen phase, and the average length of HFs (Fig. 7H and 7I). Thus, the activation of HIF-1α by DMOG recapitulated the effects of Que in promoting angiogenesis and accelerating anagen induction.

## Discussion

In this study, we demonstrate that the topical treatment of Que is effective to induce anagen entry in HF cycling and facilitate hair regrowth. Our single-cell transcriptomic landscape analysis of HFs and their surrounding niche reveals that activation of HIF-1α in EC is coupled with activated HF cycling by Que treatment to promote hair growth. Importantly, applying a HIF-1α agonist to the skin is sufficient to initiate perifollicular angiogenesis and telogen-to-anagen transition, as well as hair regrowth (Fig. 7J). Our study thus advances the understanding of hair regeneration and provides a promising therapeutic strategy against hair loss and related disorders.

Que is a dietary flavonoid abundant in various kinds of fruits and vegetables. It has been considered as a natural anti-inflammatory and anti-oxidant agent and has antiviral and antimicrobial properties (Li et al., 2021). In line with our discovery, previous studies reported that Que by intraperitoneal injections prevented/reduced alopecia areata in C3H/HeJ model and a Chinese medicine prescription containing Que stimulates HF regeneration and wound healing on burned skin (Wikramanayake et al., 2012; Zhang et al., 2020b). In addition, a glycoside form of Que, quercitrin, has been shown to increase hair growth in HF organ culture model *ex vivo* (Kim et al., 2020). Interestingly, our recent work has implicated Que as a geroprotective drug to prolong the health span in mouse models, manifested by a range of beneficial effects that alleviated tissue aging including age-related hair loss (Geng et al., 2019a). Collectively, our results suggest that Que may have potential for clinical treatment of hair loss-related disorders.

To illuminate the molecular processes involved in Que-induced hair growth, we systematically capture single-cell transcriptome profiles on different days after Que treatment. Our single-cell data reveal that early changes occurring in the Que-treated skin include angiogenesis concomitant with the expansion of endothelial cells. HFs reside in a specialized niche composed of various stromal cell types, including endothelial cells (Niemann and Watt, 2002; Hsu and Fuchs, 2012; Driskell et al., 2014). It has been noticed that the skin vasculature and various other cellular compartments undergo dramatic remodeling processes along with the HF cycle (Hsu et al., 2014a). Specifically, the extensive angiogenesis during the anagen phase has been speculated to promote hair growth via fueling oxygen, growth factors, and nutrients (Mecklenburg et al., 2000; Yano et al., 2001; Li and Tumbar, 2021). Recent work also showed that the transplantation of HF germs containing endothelial cells resulted in higher levels of HF regeneration than those without endothelial cells (Kageyama et al., 2021). Taken together, evidence from our study further supports that remodeling skin endothelial compartments can activate HFs from quiescence to the acceleration of hair regrowth.

At the molecular level, we find pronounced upregulation of the HIF-1α signaling cascade in endothelial cells upon Que treatment. Interestingly, the effect of Que on the induction of HIF-1α expression has been consistently observed in other cell types; however, their consequence on cell proliferation is cellular context-dependent, for instance, inhibiting cell proliferation in cancer cells and promoting cell proliferation in endothelial cells (Wilson and Poellinger, 2002; Lee and Lee, 2008; Bach et al., 2010; Anand et al., 2011). Particularly, we validate that one critical function of HIF-1α in endothelial cells is promoting cell proliferation and migration. Importantly, many small-molecule compounds, including the conventional tool compound DMOG used in this study, have been developed as cell-permeable and competitive inhibitors of HIF-prolyl hydroxylases, the primary enzymes for degrading HIF (Hirota, 2021). Therefore, HIF-1α activators with diverse selectivity and chemical properties have advanced to clinical studies highlighting the translational potential of our findings in repositioning these drugs for alopecia treatment and hair loss prevention.

In conclusion, we have established a dynamic single-cell transcriptome landscape of mouse hair growth triggered by Que and uncover that the HIF-1α activity-driven remodeling of vasculature plays a crucial role in orchestrating hair homeostasis and regeneration. Our findings implicate pharmacological activation of HIF-1α as a promising therapeutic intervention for hair loss in aged individuals and alopecia patients. This work provides mechanistic insights into the complex cellular and molecular interplays in a microenvironment governing the hair growth cycle and offers a valuable resource for the exploration of potential therapeutic avenues.

## Materials and methods

### Experimental animals

C57BL/6J male mice were purchased at 5 weeks of age from SiPeiFu (Beijing). Mice were housed in a controlled SPF facility and were fed a standard chow diet and provided free access to food and water throughout the study. The test compounds were dissolved in DMSO and diluted in 80% glycerin [0.3 mmol/L for Que (TCI), 160 mmol/L for Met (Torics), 30 mmol/L for GA (Selleck), and 12 mmol/L for DMOG (Millipore)]. Four days later, mice were shaved dorsally. The next day, 200 μL vehicle or compound solution was topically applied on the shaved skin every other day for 3–6 weeks at around day 42 after birth, when HFs were synchronized into the second postnatal telogen phase that lasts > 4 weeks (Muller-Rover et al., 2001; Sugaya and Hirobe, 2014; Li et al., 2020). For hair regeneration analysis in 16-month mice, 0.3 or 3 mmol/L Que was topically applied on the shaved skin every other day for 43 days. For hair regeneration analysis in young and 14-month-old aged mice, oral gavage of 0.125 or 0.625 mg/kg Que with 100 μL of 10% PEG400 in PBS was administered to young mice every other day for 2 months and to 14-month-old mice by once a week for 8 months as previously described (Geng et al., 2019a). Appearance of skin pigmentation and hair growth were monitored and documented. Scoring was done blindly.

### Cell culture

Human pMVECs were purchased form Lonza and were cultured in endothelial cell growth medium (LONZA) in 5% CO_2_ at 37°C. Cells were cultured under permissive conditions to 80% confluence, then transferred to nonpermissive conditions. After enzyme treatment, cells were collected by centrifugation and resuspended for further treatment and analysis. HEK293T cells were cultured in medium containing high-glucose DMEM (Gibco) supplemented with 10% fetal bovine serum (FBS) (Gibco), 2 mmol/L GlutaMAX (Gibco), 0.1 mmol/L NEAA (Gibco), and 1% penicillin/streptomycin (Gibco). There was no mycoplasma contamination observed during cell culture.

### Tissue sampling

Skin samples were collected and stored in ice-cold phosphate buffer saline (PBS). For cell isolation, skin tissues were minced with scissors into pieces in PBS on ice and transferred into 15 mL centrifuge tubes, rinsed twice with cold PBS and incubated at 37°C for 1 h in digestion solution (DMEM/F12 supplemented with 1 mg/mL collagenase I, 1 mg/mL collagenase IV, 1 mg/mL dispase, 0.125% trypsin-EDTA, and 2 U/mL DNase). Tissue samples were then dissociated into single-cell suspensions by pipetting, and the suspensions were passed through 40 μm strainers. The digestions were stopped by adding DMEM/F12 containing 10% FBS. Dissociated cells were collected by centrifugation at 300 × *g* for 5 min at 4°C and resuspended in 5 mL cold PBS. The cells were washed twice as mentioned above and resuspended in cold PBS supplemented with 10% FBS. The dissociated cells were then sorted by FACS (BD Influx) to remove cell debris, and excluded dead cells from the suspension of single cells by propidium iodide (PI) staining. The resultant single-cell suspension in 50 μL PBS containing 0.04% bovine serum albumin was used for 10× Genomics sequencing.

### Hematoxylin and eosin (H&E) staining

H&E staining was performed as previously described (Ma et al., 2022). The 8 μm paraffin-embedded sections were deparaffinized with xylene and rehydrated with decreasing concentration of alcohol (100%, 90%, 80%, 70%, and 50%) and water. The sections were stained in hematoxylin solution for 5 min and washed in running tap water. The sections were differentiated with 1% acid alcohol (1% HCl in 70% alcohol) for a few seconds to remove excess dye and rinsed in running water until the sections turn blue. Then the slides were counterstained with eosin for 3 min, dehydrated with increasing concentration of alcohols (50%, 70%, 80%, 90%, and 100%) and cleaned with 100% xylene, and mounted with neutral resinous mounting medium. Images were taken by the microscope. For hair cycle analysis, the individual HF in photomicrographs of H&E-stained longitudinal sections was classified based on guidelines for the accurate classification of hair cycle stages as previously described with minor modifications (Muller-Rover et al., 2001). Briefly, telogen HF is characterized by the compact ball-shaped dermal papilla that is closely attached to a small cap of hair germ. Anagen HF is defined as having an enlarged hair bulb and a thickening and prolongation of the strand of keratinocytes between the dermal papilla and the club hair.

### Immunofluorescence staining

Immunofluorescence staining was performed as previously described (Wang et al., 2020). Murine back skins were embedded in Optimal Cutting Temperature (O.C.T) compound on dry ice and stored at −80°C. 16 μm skin frozen sections were fixed in 4% paraformaldehyde for 15 min at room temperature and permeabilized with 0.4% Triton X-100 (Sigma-Aldrich) for 30 min, blocked in 10% donkey serum for 1 h, and incubated with primary antibodies at 4°C overnight. For 3D reconstruction analysis, the 80 μm skin sections were applied. After washing with PBS, the sections were incubated for 1 h with Alexa-488-conjugated secondary antibodies/Alexa-568-conjugated secondary antibodies (Jackson Immuno Research), and then the nucleus were counterstained with Hoechst 333 (Invitrogen). Images were captured by Leica 910 confocal or Zeiss LSM 900 confocal. Antibodies used for immunofluorescence staining were listed in Table S6.

### Western blot

Western blot was performed as previously described (Zhang et al., 2021b). In brief, cell samples or skin tissues were lysed in buffer containing 4% SDS and 100 mmol/L Tris-HCl (pH = 6.8) and incubated at 105°C for 10 min followed by BCA quantification of protein concentrations (BCA-02, Beijing Dingguo Changsheng biotechnology Co. Ltd). Protein lysates (20 μg per sample) were then separated by SDS-PAGE and electrotransferred to PVDF membranes (Millipore). Membranes were blocked in 5% milk, followed by incubation with primary antibodies and horseradish peroxidase-conjugated secondary antibodies. The ChemiDoc XRS+ system (Bio-Rad) was used for band visualization and the Image J software (NIH) was used for quantification analysis of protein levels. Antibodies used for Western blot were listed in Table S6.

### Quantitative RT-qPCR

Quantitative RT-PCR was performed as previously described (Ma et al., 2020). Total RNA was extracted using TRIzol reagent according to the manufacturer’s protocol. cDNA was synthesized using GoScript™ Reverse Transcription System (Promega) according to the manufacturer’s protocol from 2 μg RNA. Quantitative PCR was conducted using the iTaq Universal SYBR Green Super Mix (Bio-Rad) on a CFX384 Real-Time PCR system (Bio-Rad). The relative mRNA expression of each gene was normalized to the internal control transcript and calculated using the ∆∆Cq method with an efficiency correlation. At least three independent samples were used for RT-qPCR assays. All primers used in this study were listed in Table S7.

### Lentivirus production

The lentiviral vectors encoding HIF-1α or GAL4 were derived from the previous studies (Wang et al., 2021; Zou et al., 2021). For packaging lentiviruses, HEK293T cells were co-transfected with lentiviral expression vectors, as well as packing vectors psPAX2 and pMD2.G (Yan et al., 2019). Viral particles were collected by ultracentrifugation at 19,400 ×*g* at 4°C for 2.5 h.

### Clonal expansion assay

3,000 pMVECs were seeded in each well and cultured in endothelial cell growth medium (LONZA) for about 14 days. Afterwards, cells were fixed and stained with 0.2% crystal violet. The relative cell number was counted with Image J software.

### Cell migration assay

Cell migration assay was performed as previously described (Liu et al., 2022). The cell migration ability of pMVECs was measured by wound healing assays. Briefly, pMVECs were plated and cultured in collagen-coated 96-well plates. When the cells reached 100% confluence, a linear scratch (“wound”) was generated, and the cells were cultured and monitored by Incucyte live-cell analysis system (Essen BioScience) at 37°C for another 16 h. The number of cells that migrated into the scratch was counted using Image J software.

### Tube formation analysis

The pMVECs were resuspended in endothelial cell medium and plated in 24-well plates coated with Matrigel (BD) at a density of 6 × 10^4^ cells per well with three repeats. After 8 h of incubation, photographs were taken under an optical microscope and analyzed by Image J software.

### Flow cytometry analysis

For cell cycle analysis, 1 × 10^6^ cells were fixed in 70% pre-chilled ethanol at −20°C overnight and then incubated with staining buffer containing 0.1% Triton X-100, 0.2 mg/mL RNase A and 0.02 mg/mL PI at 37°C for 30 min. Then the cells were subjected to fluorescence-activated cell sorting (FACS) system (BD FACS Aria II) and analyzed by FlowJo software. For cellular NO production analysis, live cells were collected and stained with 1 μmol/L DAF-FM diacetate (Invitrogen) for 30 min. Experiments were performed on BD FACS Aria II and analyzed by FlowJo software.

### Droplet-based microfluidic single-cell analysis

Single cells were captured in droplet emulsions and scRNA-seq libraries were constructed according to manufacturer’s protocol using the Chromium 10× Single-Cell Instrument (10× Genomics) and 10× Genomics Chromium Single Cell 3ʹ GEM Library and Gel Bead Kit v3. In brief, cells were loaded in each channel with a target output of 8,000 cells per sample and appropriate cell concentration was measured by Moxi GO II (Orflo Technologies). All reactions were performed in the Bio-Rad C1000 Touch Thermal cycler with 96 Deep-Well Reaction Module in which 12 cycles were used for cDNA amplification and sample identification. Amplified cDNAs and final libraries were then evaluated on a Fragment Analyzer (AATI) using a High Sensitivity NGS Analysis Kit (Advanced Analytical). The average fragment length of the 10× cDNA libraries was assessed with the AATI, and quantified by qPCR using the Kapa Library Quantification kit. All the libraries were diluted to a final concentration of 2 nmol/L and pooled together for each run of NovaSeq sequencing. All the libraries were sequenced on the NovaSeq 6000 Sequencing System (Illumina).

### Processing raw data from scRNA-seq of 10× Genomics

Single-cell gene expression was analyzed using the Cell Ranger Single Cell Software Suite (V 3.1.0) (10× Genomics) to perform quality control including sample de-multiplexing, barcode processing, and single-cell gene counting. First, the sample-specific FASTQ files were aligned to the mouse reference genome (mm10) with “*cellranger count*” and the quality assessment was performed with default parameters. Then, a digital gene expression matrix was generated using STAR aligners. The filtered gene expression matrix was used for downstream analyses.

### scRNA-seq data analysis and identification of cell type markers

The resulting gene-barcode matrix was imported into Seurat (version 3.2.0) (Stuart et al., 2019) for quality control, dimensionality reduction, cell clustering, and differential expression analysis. Specifically, quality control metrics included the number of genes between 500 and 5,000 genes per cell, and no more than 20% mitochondrial genes. DoubletFinder (version 2.0.3) software was used to predict and remove the influence of technical artifacts known as “doublets”. Finally, 35,358 cells were remained for downstream bioinformatic analyses. To better eliminate the false positives of biological heterogeneity caused by technical factors such as sequencing depth in scRNA-seq data, we used SCTransform (version 0.3.2) (Hafemeister and Satija, 2019) to normalize and scale the matrix. The “*PrepSCTIntegration*” and “*FindIntegrationAnchors*” functions were used to select integration anchors and perform downstream integration. These anchors were then used to integrate the dataset of all samples with “*IntegrateData*” function. The integrated data were then used for cell clustering and visualization with Seurat. First, principal component analysis (PCA) dimensions were calculated with the “*RunPCA*” function. Next, unsupervised clustering of the data was performed with the “*FindNeighbors*” and “*FindClusters*” functions. For the “*FindNeighbors*” function, we used the top 30 principal component dimensions to construct a Shared Nearest Neighbor (SNN) Graph for our datasets. Then, we clustered the cells with the function “*FindClusters*” using a SNN modularity optimization-based clustering algorithm with a resolution of 2.0. Finally, for visualization, we used the “*RunUMAP*” function with default parameters and top 30 principal component dimensions. To identify genes with enriched expression in each cell type, we used the “*FindAllMarkers*” function in the integrated dataset which applies a Wilcoxon Rank Sum test to identify the markers for each cell type (see also Table S1).

### Identification of time-dependent TDEGs

To identify TDEGs, we were inspired by the “*plot_pseudotime_heatmap’*’ function in the R package Monocle2 (version 2.14.0) (Qiu et al., 2017) and customized a function based on it. First, the function of “*FindMarkers*” in Seurat was used to identify DEGs between two time points (D0, D3, D9, and D15) in each cell type (see also Table S2). Only those genes with “|avg_logFC|” > 0.25 and “*p*_val_adj” < 0.05 were considered as HF regeneration-related DEGs. Second, for each cell type, we used the above genes to construct an expression matrix (genes as rows, cells as columns), and then sort all cells by time scale (i.e., each column in the expression matrix was ranked according to the time rank corresponding to each cell). Next, we used the “*genSmoothCurves*” function to fit smooth spline curves for the gene expression matrix dynamics along time in a gene-wise manner and return the corresponding response matrix. Finally, the “ward.D2” method was used to cluster the matrix by row hierarchical clustering through the “*hclust*” function, and the clustering results were assigned to 4 groups (see also Table S3). The R package pheatmap (version 1.0.12) and ggplot2 (version 3.3.3) were used to visualize the time-dependent genes expression pattern.

### GO analysis

GO analysis of DEGs was performed by Metascape (Zhou et al., 2019) and visualized with the ggplot2 R package (version 3.3.3). Representative terms selected from the top 100 ranked GO terms or pathways (*P* < 0.01) were displayed.

### Gene set enrichment analysis (GSEA)

Gene set enrichment analysis was performed using GSEA software (Mootha et al., 2003; Subramanian et al., 2005) and R software. Gene sets were obtained or compiled from the MSigDB database, Kyoto Encyclopedia of Genes and Genomes (KEGG) (Kanehisa and Goto, 2000), and Mouse Genome Informatics (MGI) (Blake et al., 2021).

### Pseudotime trajectory analysis

Developmental pseudotime trajectories of mouse HF cell were reconstructed using the R package Monocle2 (version 2.14.0) (Qiu et al., 2017). Genes for subsequent clustering were marked using the “*setOrderingFilter*” function, and the marker genes of HF subtypes identified in the above “scRNA-seq analysis” were set as “ordering_genes”. Dimensionality reduction analysis was performed using the “*reduceDimension*” function, and the “method” was set to “DDRTree”. The cell trajectory was then captured using the “*orderCells*” function, with the outerBulge cluster was set as “root_state”. The function of “*differentialGeneTest*” was used to identify genes that were significantly covariant with pseudotime time. Benjamini-Hochberg multiple test correction was used to calculate FDR, and genes with “*q*-value” < 0.05 and expression ratio > 10% were considered to vary significantly with pseudotime.

### Cell–cell communication analysis

To identify and visualize cell–cell interaction strength between vehicle- and Que-treated groups, we used the R package CellChat (version 1.1.3) (Jin et al., 2021), an algorithm for analyzing cell–cell communication at the single-cell level. Briefly, we followed the official workflow to load the expression matrix normalized by the Seurat workflow and metadata of the vehicle- and Que-treated groups into CellChat. Then a CellChat software object was created and preprocessed using a workflow with standard parameters. A total of 2,021 mouse ligand-receptor interaction pairs including secrete autocrine/paracrine signaling interactions, ECM-receptor interactions, and cell–cell contact interactions were used for cell–cell communication analysis. The vehicle- and Que-treated groups were merged by the “*mergeCellChat*” function. Finally, potential ligand-receptor interactions that were significantly differentially expressed between the vehicle- and Que-treated groups were calculated based on the ligand-receptor interaction database as described above.

### Gene set score analysis

To score individual cell for pathway activities, we used the R package AUCell (version 1.8.0) (Aibar et al., 2017). The canonical pathways were downloaded from the MsigDB database, KEGG database, and MGI (see also Table S4). These gene sets were used to score each cell. We used an expression matrix to calculate gene expression rankings in each cell with the “*AUCell_buildRankings*” function. Area-under-the-curve (AUC) values were calculated (“*AUCell_calcAUC*” function) based on gene expression rankings.

### RNA-seq library construction and sequencing

RNA-sequencing library construction and sequencing was performed as previously described (Ma et al., 2020). Total RNA was extracted from 1 × 10^6^ cells using Trizol according to the manufacturer’s instructions. The quality and quantity of total RNA were assessed by Fragment Analyzer (AATI) and NanoDrop ND-1000 (Wilmington), respectively. The mRNA was isolated from 2 μg of total RNA using the NEBNext Poly (A) mRNA Magnetic Isolation Module. Subsequently, isolated mRNA was used for RNA library construction using NEBNext Ultra RNA library prep kit for Illumina. The generated libraries were pooled and sequenced on Illumina HiSeq 4000 platforms with paired-end 150-bp sequencing.

### RNA-seq data analysis

Trim Galore (version 0.4.5) software was used for automate adapter trimming and quality control, and Hisat2 (version 2.0.4) (Kim et al., 2015) with default parameters was used to map the cleaned reads to the UCSC mm10 mouse genome. HTSeq (version 0.6.1) (Anders et al., 2015) software was used to count the number of reads mapped in each annotated gene based on the mapping results. R package DESeq2 (version 1.2.4) (Love et al., 2014) was used to calculate DEGs with the cutoff values of Benjamini-Hochberg adjusted *P* value (“*p*.adjust”) < 0.05 and “|log_2_ (fold change)|” > 0.58 (see also Table S1).

### Statistical analysis

The statistical analysis was performed and analyzed by two-tailed unpaired Student’s *t*-test or two-way ANOVA with Sidak’s test in Graphpad Prism 8.0 software. Data are presented as the mean ± SEMs and *P* values are presented in indicated figures as appropriate.

## Supplementary Material

pwac062_suppl_Supplementary_Materials

pwac062_suppl_Supplementary_Table_S1

pwac062_suppl_Supplementary_Table_S2

pwac062_suppl_Supplementary_Table_S3

pwac062_suppl_Supplementary_Table_S4

pwac062_suppl_Supplementary_Table_S5

pwac062_suppl_Supplementary_Table_S6

pwac062_suppl_Supplementary_Table_S7

## Data Availability

The raw sequence data reported in this paper have been deposited in the Genome Sequence Archive in National Genomics Data Center, China National Center for Bioinformation/Beijing Institute of Genomics, Chinese Academy of Sciences (GSA: CRA006512).

## Abbreviations

BC: basal cell
BVE: blood vascular endothelial cell
DEGs: differentially expressed genes
DMOG: dimethyloxallyl glycine
EC: endothelial cell
GA: gallic acid
GC: granular cell
GL: germlayer cell
GO: Gene Ontology
HF: hair follicle
HFSC: hair follicle stem cell
HG: hair germ cell
HS1: hair shaft cell 1
HS2: hair shaft cell 2
HFB: hair follicle bulge cell
innerBulge: inner bulge cell
IRS: inner root sheath cell
LC: Langerhans cell
LE: lymphatic endothelial cell
Met: metformin
MC: mitosis basal cell
migraBulge: migrating bulge cell
Mac1: macrophage 1
Mac2: macrophage 2
NO: nitric oxide
outerBulge: outer bulge cell
PHD: prolyl hydroxylase
pMVEC: human primary skin microvascular endothelial cells
Que: quercetin
SC: spinous cell
SG: sebaceous gland cell
SM: smooth muscle cell
SNN: shared nearest neighbor
TAC: transit-amplifying cell
TC: T cell
TDEGs: differentially expressed genes along the time trajectory
t-SNE: t-distributed stochastic neighbor embedding
UHF: upper hair follicle cell
UMAP: uniform manifold approximation and projection
upBulge: upper bulge cell
