# Correction to: Single-cell profiling reveals a potent role of quercetin in promoting hair regeneration

**DOI:** 10.1093/procel/pwaf035

**Published:** 2025-05-20

This is a correction to: Qian Zhao, Yandong Zheng, Dongxin Zhao, Liyun Zhao, Lingling Geng, Shuai Ma, Yusheng Cai, Chengyu Liu, Yupeng Yan, Juan Carlos Izpisua Belmonte, Si Wang, Weiqi Zhang, Guang-Hui Liu, Jing Qu, Single-cell profiling reveals a potent role of quercetin in promoting hair regeneration, *Protein & Cell*, Volume 14, Issue 6, June 2023, Pages 398–415, https://doi.org/10.1093/procel/pwac062.

During a recent review of this article, the authors identified an inadvertent misplacement of an image in Fig. 7I resulting from confusion in image grouping. The corrected figure is shown below. This correction does not affect the study’s conclusions or overall discussion.


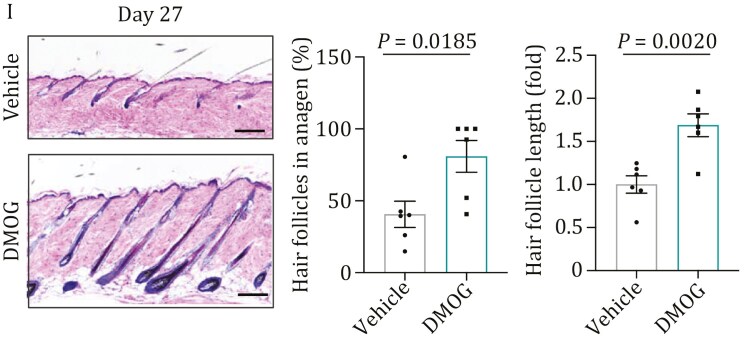


These details have been corrected only in this correction notice to preserve the published version of record.

