# Supplementary material for: Single-cell profiling reveals a potent role of quercetin in promoting hair regeneration
Source: Protein Cell. 2022 Nov 30;14(6):398–415. doi: 10.1093/procel/pwac062 (PMC10246722; doi:10.1093/procel/pwac062)
Supplement: pwac062_suppl_Supplementary_Materials [file pwac062_suppl_supplementary_materials.pdf]

Figure S1. Phenotypical analyses of mice treated with Que.

(A) Left, representative hair coats of mice topically treated with vehicle, 0.1 mmol/L Que, 0.3 mmol/L Que, 3 mmol/L Que or 30 mmol/L Que on day 29 post-treatment. Right, quantification for appearance of melanin pigmentation in mouse skin treated with vehicle, 0.1 mmol/L Que, 0.3 mmol/L Que, 3 mmol/L Que or 30 mmol/L Que. Pigmentation scoring was described in Materials and methods. Vehicle, n = 8; 0.1 mM Que, n = 8; 0.3 mM Que, n = 8; 3 mM Que, n = 8; 30 mM Que, n = 8. Two-way ANOVA with Sidak's test was used and data are presented as the mean ± SEMs.

(B) Left, representative hair coats of oral gavage of vehicle, 0.125 or 0.625 mg/kg treated mice on day 54 post-treatment. Right, quantification for appearance of melanin pigmentation in mouse skin treated with vehicle, 0.125 or 0.625 mg/kg Que. Vehicle, n = 7; 0.125 mg/kg Que, n = 14; 0.625 mg/kg Que, n = 7. Two-way ANOVA with Sidak's test was used and data are presented as the mean ± SEMs.

(C) Immunostaining of Ki67 of skin tissue sections on young mice after oral gavage with vehicle or 0.625 mg/kg Que. Right, the quantitative data of Ki67-positive cells per HF in vehicle- or Que-treated groups. Vehicle, n = 6; Que, n = 6 and 15 HFs were quantified for each individual. Two-tailed unpaired Student's t-test was used and data are presented as the mean ± SEMs. Scale bars, 100 μm.

(D) Immunostaining of CD31 of skin tissue sections on young mice after oral gavage with vehicle or 0.625 mg/kg Que. Right, the quantitative data of CD31-positive area in vehicle- or Que-treated groups. Vehicle, n = 6; Que, n = 6. Two-tailed unpaired Student's t-test was used and data are presented as the mean ± SEMs. Scale bars, 100 μm.

(E) Left, H&E staining of skin tissues sections on 22-month mice post-oral gavage with vehicle or Que. Right, quantitative data of HF length and the percentage of HFs in anagen in vehicle- or Que-treated groups. Vehicle, n = 4; Que, n = 4 and 20 HFs were quantified for each individual. Two-tailed unpaired Student's t-test was used and data are presented as the mean ± SEMs. Scale bars, 200 μm.

(F) Immunostaining of CD31 of skin tissues sections on 22 months mice after oral gavage with vehicle or Que. Right, the quantitative data of CD31-positive area in vehicle- or Que-treated groups. Vehicle, n = 4; Que, n = 4. Two-tailed unpaired Student's t-test was used and data are presented as the mean ± SEMs. Scale bars, 100 μm.

Figure S2. Single-cell transcriptomic profile analyses of different cell types with Que treatment.

(A) The distribution of quality controls, including the median number of genes, sequencing saturation, and mapping rate in the scRNA-seq data of each sample.

(B) Scatter plot showing the proportion of mitochondrial genes for each cell.

(C) Histograms showing the number of genes (left) and unique molecular identifiers (UMIs) (right) detected in individual cells.

(D) Boxplot showing the value of ROGUE (a method for accurately quantify the purity of an identified cell type) of each cell type. Box shows the median and the quartile range (25-75%) and the length of whiskers represents 1.5× IQR.

(E) UMAP plots showing the cell distribution of all samples. Cells are marked by samples (left) and groups (right).

(F) UMAPs plots showing the cell distribution of each sample individually.

(G) Left, heatmap showing row z-score expression of top 50 cell type-specific genes. Right, enriched GO terms and pathways for marker genes. Abbreviations for each ell type are the same as those in Figure 2B.

(H) UMAP plots showing the expression levels of *Lef1* and *Mki67*. The threshold is shown on the bottom.

(I) Circular plots showing the number and proportion of TDEGs for different cell types in each module. Cell type acronyms are the same as in Figure 2B.

(J) Circular plots showing the TDEGs shared by at least two cell types (Frequency $\geq$ 2) in module 2 and module 3.

Figure S3. Transcriptional profiles of HF subclusters upon Que treatment.

(A) Dot plot showing the expression levels of representative genes for each HF sub-cell types.

(B) Violin plots showing the expression levels of HFSC marker genes in different HF subclusters.

(C) Quiescence scores of HF sub-cell types calculated by expression levels of *Foxc1* target genes.

(D) Heatmaps showing the expression profiles of transcriptional factors (TFs) along with the pseudotime, which were divided into three clusters with the expression pattern. Representative TFs are listed on the right.

(E) Heatmap showing the gene set scores of signaling pathway related to hair growth in different HF subclusters with Que treatment.

Figure S4. Single-cell transcriptional analyses of vascularization in response to Que treatment.

(A) UMAP plot showing the distribution of endothelial cells. BE, blood vascular endothelial cell; LE, lymphatic endothelial cell.

(B) UMAP plots showing the expression levels of representative marker genes of BVE and LE.

(C) The bar plot showing the differentially expressed signaling pathways identified by the differences in the information flow of each signaling pathway between the Que and vehicle group in EC and GL.

(D) 3D reconstruction of immunofluorescence staining of VWF in HFs on day 3 post-treatment with vehicle or Que. Right, the quantitative data of VWF-positive area in the skin. Vehicle, n = 6; Que, n =6. Two-tailed unpaired Student's t-test is used and data are presented as the mean ± SEMs. Scale bars, 50 μm.

(E) Heatmaps showing the expression profiles of genes involving in angiogenesis (left), migration (middle), and endothelial cell proliferation (right) pathways.

(F) Violin plots showing the transcriptional levels of *Hif-1a* in BVE and LE in vehicle and Que groups.

(G) Western blot analysis of HIF-1α protein expression levels in skin tissues treated with vehicle or Que. Vehicle, n = 6; Que, n = 6. Two-tailed unpaired Student's t-test was used and data are presented as the mean ± SEMs.

(H) Western blot analysis of HIF-1α protein expression levels in in skin tissues treated with vehicle or DMOG. Vehicle, n = 6; DMOG, n = 6. Two-tailed unpaired Student's t-test was used and data are presented as the mean ± SEMs.

TABLE LEGENDS

Table S1. Marker genes for each cell type in mouse skin.

Table S2. Pairwise differentially expressed genes (PDEGs) for each cell type.

Table S3. Dynamic differentially expressed genes along the time trajectory (TDEGs) for each cell type.

Table S4. Gene sets used in this study.

Table S5. Differentially expressed genes (DEGs) in bulk RNA-seq data.

Table S6. Antibodies used in this study.

Table S7. Primers used in this study.

Figure S1

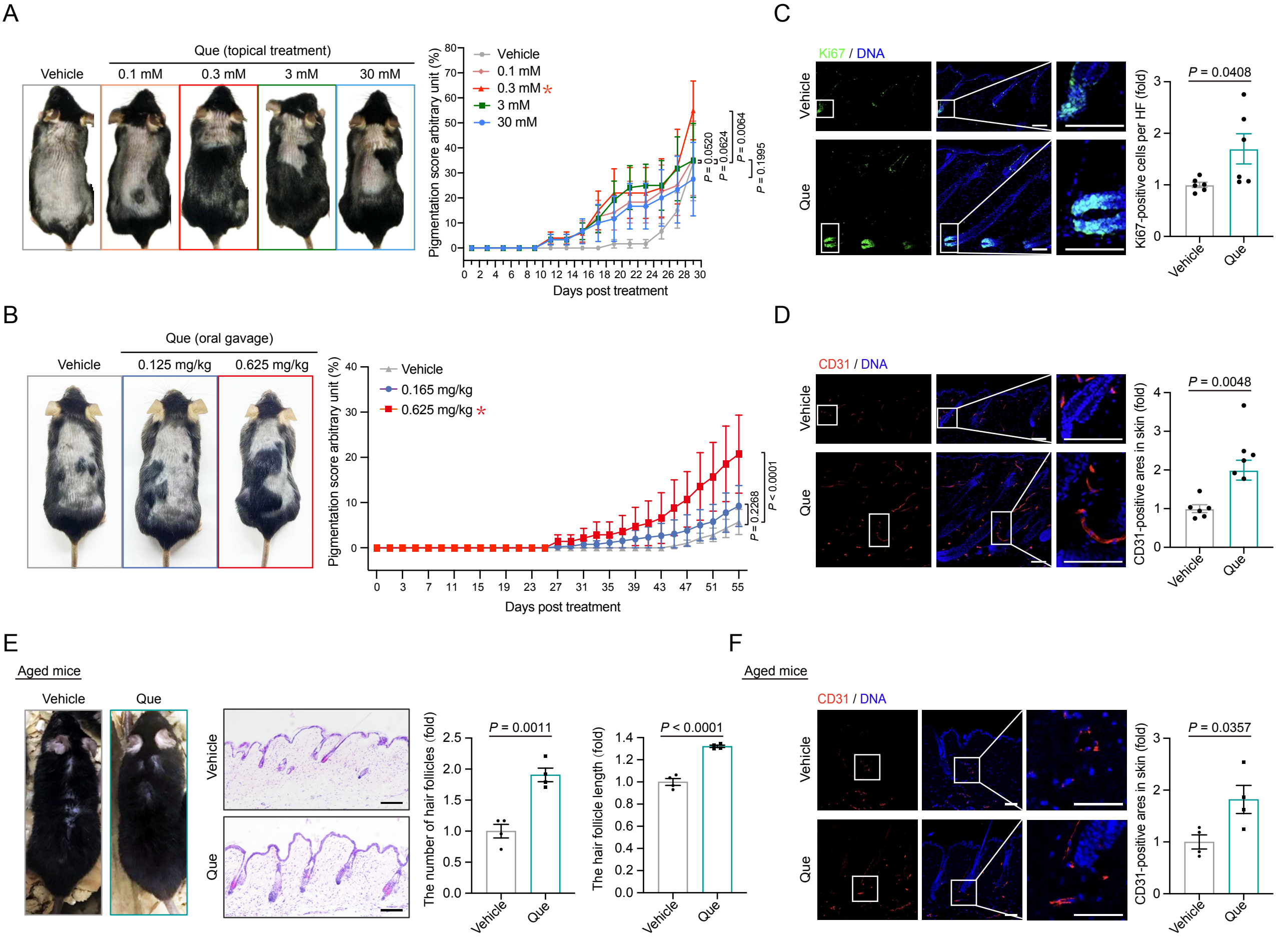

A
Que (topical treatment)
Vehicle
0.1 mM
0.3 mM
3 mM
30 mM
Pigmentation score arbitrary unit (%)
Days post treatment
P = 0.0520
P = 0.0624
P = 0.0064
P = 0.1995
C
Ki67 / DNA
Vehicle
Que
Ki67-positive cells per HF (fold)
P = 0.0408
B
Que (oral gavage)
Vehicle
0.125 mg/kg
0.625 mg/kg
0.165 mg/kg
P = 0.2268
P < 0.0001
D
CD31 / DNA
CD31-positive ares in skin (fold)
P = 0.0048
E
Aged mice
The number of hair follicles (fold)
P = 0.0011
The hair follicle length (fold)
P < 0.0001
F
Aged mice
CD31 / DNA
CD31-positive ares in skin (fold)
P = 0.0357

Figure S2

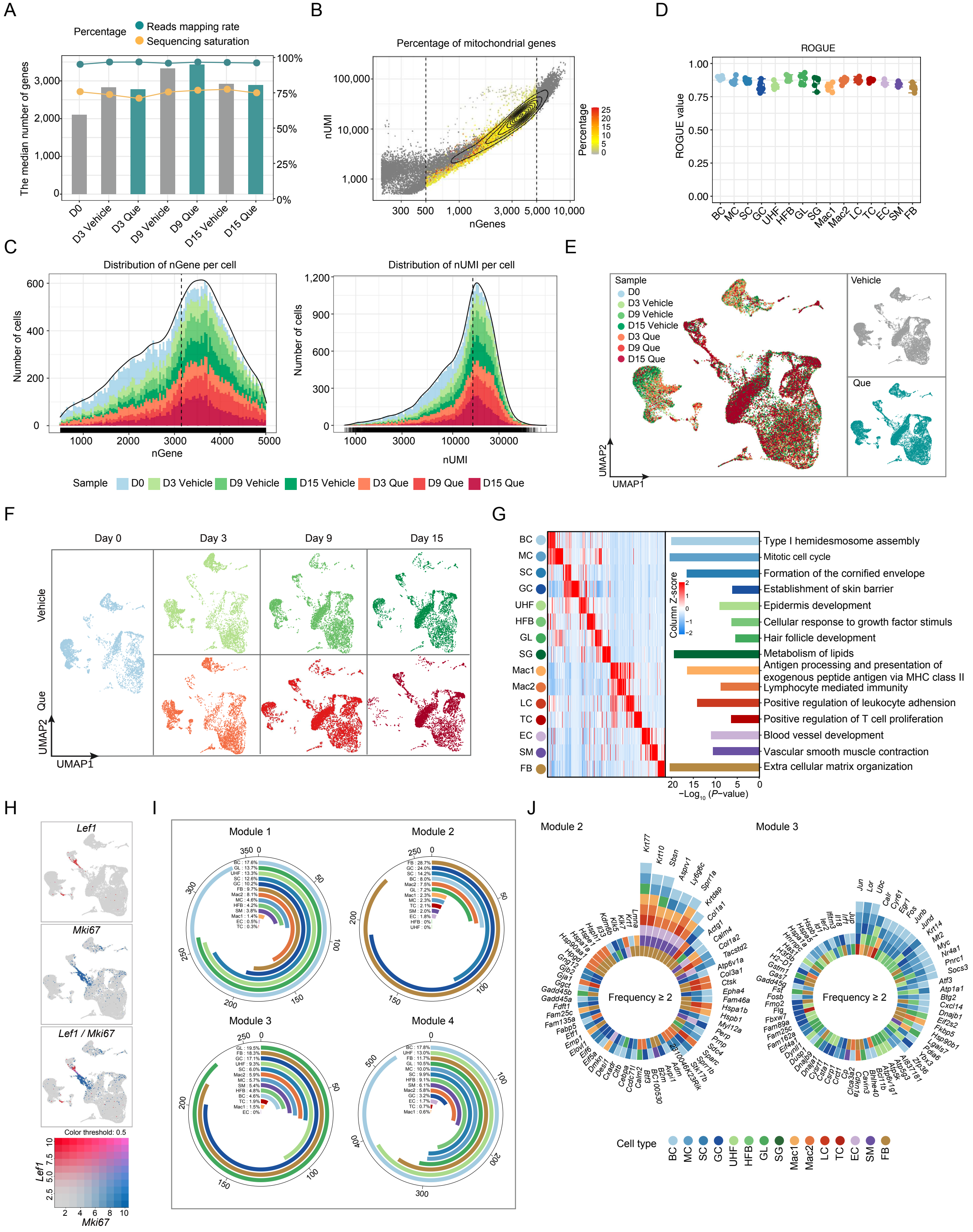

A
Percentage
Reads mapping rate
Sequencing saturation
The median number of genes
D0
D3 Vehicle
D3 Que
D9 Vehicle
D9 Que
D15 Vehicle
D15 Que
B
Percentage of mitochondrial genes
nUMI
nGenes
Percentage
C
Distribution of nGene per cell
Number of cells
nGene
Distribution of nUMI per cell
nUMI
Sample
D
ROGUE
ROGUE value
BC
MC
SC
GC
UHF
HFB
GL
SG
Mac1
Mac2
LC
TC
EC
SM
FB
E
Sample
Vehicle
Que
UMAP1
UMAP2
F
Day 0
Day 3
Day 9
Day 15
G
Column Z-score
Type I hemidesmosome assembly
Mitotic cell cycle
Formation of the cornified envelope
Establishment of skin barrier
Epidermis development
Cellular response to growth factor stimuls
Hair follicle development
Metabolism of lipids
Antigen processing and presentation of exogenous peptide antigen via MHC class II
Lymphocyte mediated immunity
Positive regulation of leukocyte adhesion
Positive regulation of T cell proliferation
Blood vessel development
Vascular smooth muscle contraction
Extra cellular matrix organization
−Log10 (P−value)
H
Lef1
Mki67
Lef1 / Mki67
Color threshold: 0.5
I
Module 1
Module 2
Module 3
Module 4
J
Module 2
Module 3
Frequency ≥ 2
Cell type

Figure S3

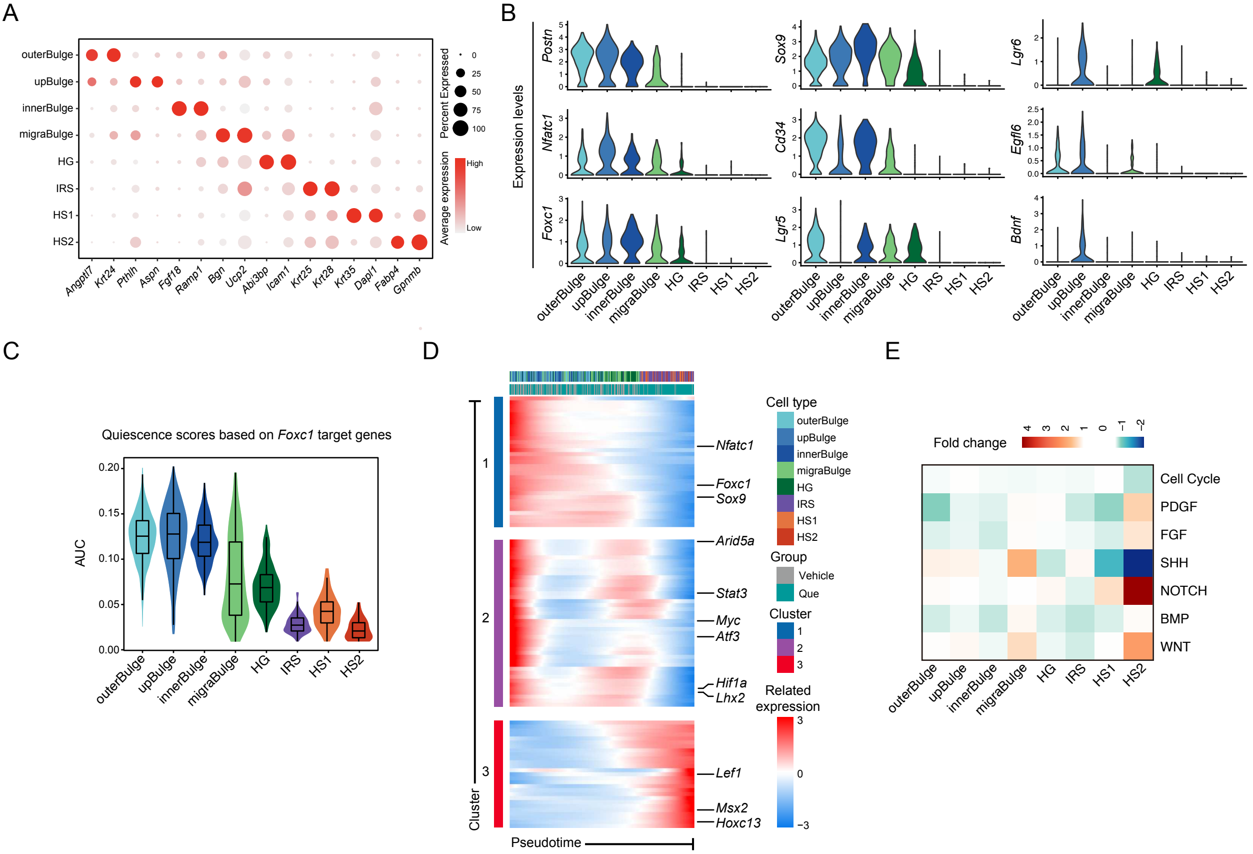
A
B
C
D
E
Percent Expressed
Average expression
High
Low
Expression levels
Quiescence scores based on Foxc1 target genes
AUC
Cell type
outerBulge
upBulge
innerBulge
migraBulge
HG
IRS
HS1
HS2
Group
Vehicle
Que
Cluster
Related expression
Pseudotime
Nfatc1
Foxc1
Sox9
Arid5a
Stat3
Myc
Atf3
Hif1a
Lhx2
Lef1
Msx2
Hoxc13
Fold change
Cell Cycle
PDGF
FGF
SHH
NOTCH
BMP
WNT

Figure S4

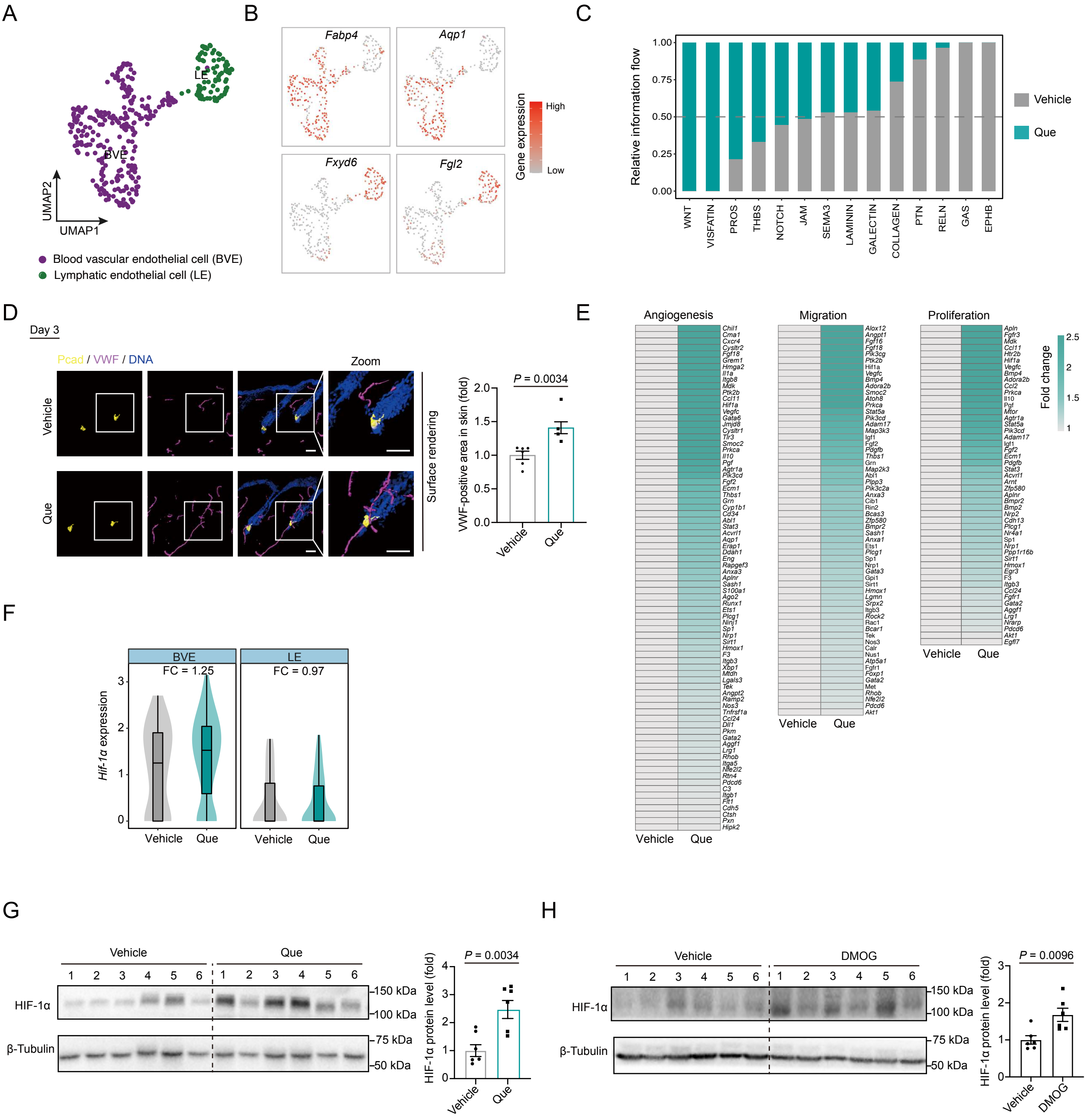

A
B
C
LE
BVE
UMAP2
UMAP1
Blood vascular endothelial cell (BVE)
Lymphatic endothelial cell (LE)
Fabp4
Aqp1
Fxyd6
Fgl2
Gene expression
High
Low
Relative information flow
1.00
0.75
0.50
0.25
0.00
WNT
VISFATIN
PROS
THBS
NOTCH
JAM
SEMA3
LAMININ
GALECTIN
COLLAGEN
PTN
RELN
GAS
EPHB
Vehicle
Que
D
Day 3
Pcad / VWF / DNA
Zoom
Vehicle
Que
Surface rendering
P = 0.0034
VWF-positive area in skin (fold)
2.0
1.5
1.0
0.5
0.0
E
Angiogenesis
Migration
Proliferation
Fold change
2.5
2
1.5
1
F
BVE
LE
FC = 1.25
FC = 0.97
Hif-1α expression
3
2
1
0
G
H
HIF-1α
β-Tubulin
150 kDa
100 kDa
75 kDa
50 kDa
DMOG
P = 0.0096
HIF-1α protein level (fold)
4
3
2
1
0
